# Fine-Scale Cartography of Human Impacts along French Mediterranean Coasts: A Relevant Map for the Management of Marine Ecosystems

**DOI:** 10.1371/journal.pone.0135473

**Published:** 2015-08-12

**Authors:** Florian Holon, Nicolas Mouquet, Pierre Boissery, Marc Bouchoucha, Gwenaelle Delaruelle, Anne-Sophie Tribot, Julie Deter

**Affiliations:** 1 Andromède Océanologie, 7 place Cassan, 34280 Carnon, France; 2 Institut des Sciences de l’Evolution (ISEM)—UMR 5554 CNRS—IRD—UM, Campus de l’Université de Montpellier, 34095 Montpellier cedex 5, France; 3 Agence de l’Eau Rhône-Méditerranée-Corse, Délégation de Marseille, Immeuble le Noailles, 62 La Canebière, 13001 Marseille, France; 4 Laboratoire Ifremer Environnement Ressources Provence-Azur-Corse, Centre Méditerranée—Zone Portuaire de Brégaillon—CS20 330–83507 La Seyne-sur-Mer Cedex, France; Università di Genova, ITALY

## Abstract

Ecosystem services provided by oceans and seas support most human needs but are threatened by human activities. Despite existing maps illustrating human impacts on marine ecosystems, information remains either large-scale but rough and insufficient for stakeholders (1 km² grid, lack of data along the coast) or fine-scale but fragmentary and heterogeneous in methodology. The objectives of this study are to map and quantify the main pressures exerted on near-coast marine ecosystems, at a large spatial scale though in fine and relevant resolution for managers (one pixel = 20 x 20 m). It focuses on the French Mediterranean coast (1,700 km of coastline including Corsica) at a depth of 0 to 80 m. After completing and homogenizing data presently available under GIS on the bathymetry and anthropogenic pressures but also on the seabed nature and ecosystem vulnerability, we provide a fine modeling of the extent and impacts of 10 anthropogenic pressures on marine habitats. The considered pressures are man-made coastline, boat anchoring, aquaculture, urban effluents, industrial effluents, urbanization, agriculture, coastline erosion, coastal population and fishing. A 1:10 000 continuous habitat map is provided considering 11 habitat classes. The marine bottom is mostly covered by three habitats: infralittoral soft bottom, *Posidonia oceanica* meadows and circalittoral soft bottom. Around two thirds of the bottoms are found within medium and medium high cumulative impact categories. Seagrass meadows are the most impacted habitats. The most important pressures (in area and intensity) are urbanization, coastal population, coastal erosion and man-made coastline. We also identified areas in need of a special management interest. This work should contribute to prioritize environmental needs, as well as enhance the development of indicators for the assessment of the ecological status of coastal systems. It could also help better apply and coordinate management measures at a relevant scale for biodiversity conservation.

## Introduction

Oceans and seas are very important for human well-being; their ecosystems provide among the most important ecosystem services: provision of food, natural shoreline protection against storms and floods, water quality maintenance, support of tourism and other cultural benefits, and maintenance of basic global life support systems [1]. The challenge lies in keeping these resources in a sustainable state of use, which is the main objective of the European Union's Marine Strategy Framework Directive (MSFD, 2008/56/EC) by achieving Good Environmental Status (GES) of EU's marine waters. Yet marine ecosystems and marine resources are under severe anthropogenic threats: population growth, land use change and habitat loss, overfishing and destructive fishing methods, illegal fishing, invasive species, climate change, pollution, increased demand for food and a shift in food preference [2]. The human impact is so great that no region can be considered virgin territory [3–5]. Protecting marine biodiversity and the essential ecosystem services it supports is considered a top priority by different authorities: the scientific community, resource managers, national and international policy agreements, including the MSFD and the Convention on Biological Diversity [6]

In this context, it is essential to analyze species and habitat distribution, environmental variables and human threats but also their correlations. Spatial distribution of anthropogenic pressures is particularly important because it is the basis of numerous other studies: ecological indicators development, species distribution analysis, design of marine reserves and of conservation plans. In this context, large-scale (continental, worldwide) studies are now commonly conducted while local studies (regional) are lacking [7–9]. Naturally, generalization often leads to an extrapolation of the spatial and temporal scales at which reliable predictions can be made because by definition large-scale models are not able to fully account for fine-grained complexity [10,11]. Moreover, large-scale predictions and their limitations may be particularly hard to understand and to use for regional managers and local policy makers focusing on specific interests (i.e. < 1km² grid cells). There is thus a paradox between the international scale of political will and the local scale of biodiversity conservation, but also a gap between global analyses and what can really be done in the field [12]. Consequently, there is a need to provide managers and stakeholders with local fine-scale information.

In order to fill this gap, fine-scale mapping efforts are multiplying in Europe especially in France [13–15], Spain [16–18], Italy [12,19–21] or Greece [22,23] or along the Baltic sea [24]. Because of the high costs to acquire such data, these fine-scale maps are generally funded in order to respond to specific and local objectives (the study of protected areas, a specific habitat [25] or particular features [26], environmental impact assessment [27]). Consequently, they mostly remain local (often a bay) and thus fragmentary (in space but also for the considered habitats and/or pressures) and heterogeneous in their methodology [12]. Moreover they are often available with difficulty: grey literature [15] or communications during conferences [28] instead of publications (but see [29]). All of this can be an obstacle to understand the impact of pressures on coastal marine habitats and thus to make decision at a local and regional scale. Fine-scale (15 x 15 m grid cells) spatial models have been recently developed in order to link multiple pressures with various coastal ecosystem status within a marine protected area [30]. The implementation into geographical information systems (GIS) allows a predictive approach of the consequences of different management alternatives [30–32]. This represents an important decision support tool for choosing efficient management solutions in the face of complex interactions and high uncertainty. Information concerning pressure distribution (presence/absence of relevant human activities, weighted distance of these activities) have been here successfully used in order to map potential impacts [33–36]. These data are so useful for local managers that they should exist all along the coast. A map of the diverse coastal marine habitats, of coastal pressures and impacts on these habitats extended to the entire coast would have an interest for the local managers and stakeholders but also for regional and national authorities. It would permit to feed the overall think on the coastal use but also highlight conservation and management priorities, compare different sites and management ways and assess the water body quality.

The objective of this work is to map and quantify, at a large spatial scale though in fine and relevant resolution for managers, the main drivers and pressures triggering changes within coastal marine ecosystems. In order to reach these objectives, localization of the different pressures exerted and their impacts is needed as much as maps of marine ecosystems. Among numerous seas listed on Earth, Mediterranean presents the particularity of being a biodiversity hotspot facing numerous and strong threats [37–40]. While maps of cumulative human impacts on marine ecosystems exist at the scale of the Mediterranean and Black sea and even at worldwide scale [5,40–42], this information could be completed along the coast. For instance, the resolution used by Micheli et al. [41] within the Mediterranean and Black sea is 1 km² pixels and no data is associated with the first pixels close to the coast, where most anthropogenic pressures are concentrated.

Interested in data that could be of real use to local managers and stakeholders, we sampled data from a homogeneous environmental policy context and thus focused on a unique country: France with its 1700 km of Mediterranean coastline, including Corsica. Our goals were to (1) provide the first complete marine coastal habitat map of the French Mediterranean coast (including Corsica), and (2) to quantify and map cumulative impacts to provide the data needed (one pixel = 20 x 20 m) to help the development of an effective marine policy. On these bases, we identified the most and least impacted areas (water bodies), the top threats affecting coastal waters, and the areas representing top priorities for ecosystem-based management and conservation efforts. The cumulative impact map obtained will be useful for local decision makers and thus complementary to large-scale previous works [41].

## Materials and Methods

### Marine habitats

The study considers the entire French Mediterranean coastline (including Corsica) included within the 46 water bodies of homogeneous water according to the Water Framework Directive (WFD,2000/60/EC) [43]. Interested in costal-based impacts, we particularly focus on the shallow part: between 0 and -80 m. After a bibliographic synthesis, we gathered and homogenized data on habitat maps; these data were collected by Andromède Océanologie, Agence de l'Eau RMC;Conservatoire du Littoral, DREAL PACA; EGIS EAU, ERAMM, GIS POSIDONIE, IFREMER, Institut océanographique Paul Ricard, Nice Côte d'Azur, TPM, Programme CARTHAM—Agence des Aires Marines Protégées, ASCONIT Consultants, COMEX-SA, EVEMAR, IN VIVO, Sintinelle, Stareso, Programme MEDBENTH, Université de Corse (EQEL), Ville de St Cyr-sur-mer, Ville de Cannes, Ville de Marseille, Ville de St Raphaël, Ville de St Tropez (S1–S3 Figs).

Gaps were completed with the program DONIA [44] with a fine scale (1:10 000 map) between 0 and -80 m and a lower resolution (1:25 000) beyond (S1–S3 Figs). Campaigns were led between 2010 and 2014 using first aerial or satellite photography (in order to measure the spatial extent of habitats in shallow waters) and a multi-beam echo-sounder GeoSwath Plus (Kongsberg Geoacoustics LTD) survey (to draw the bathymetry). Then, a side-scan sonar survey (used in more turbid and deeper (< -15 m) waters) was led. By ensonifying a swath of seabed and measuring the amplitude of the backscattered return signals, an image of the seabed was built up with information on the morphology and substrate content. We used a Klein System 3900 with a frequency comprised between 445 and 900 kHz. After that, sonar information was post-treated to determine the potential presence and coverage of underwater habitat representation. All of these data allow achieving a preliminary cartography of benthic habitats.

Numerous uncertainties still remained after this preliminary cartography work. Direct observations (“ground-truth points”) were thus needed through diving sessions (around 1600 dives between 0 and -80 m all along the coastline between 2010 and 2014). They included classic dives and “towed dives” that allowed the sampling of 20 920 ground-truth points. During “towed dives”, the diver was actively able to maneuver a “towboard” to maintain a relative constant elevation above the seabed. The towboard was equipped with an underwater GPS transducer providing the accurate position and exact depth of the diver in real-time to the surface operator. The diver equipped with an integrated communication system transmitted a large quantity of information on benthic habitats (community of organisms which lived on, in, or near the seabed, state of the habitat, occurrence of impacts on the habitat). Occasional exploring dives aimed, by means of *in situ* observation, to clarify data. These dives allowed to recognize the nature of the seabed and to characterize benthic populations. Field work was organized in cooperation with the French water agency (public authority) which gave permission to conduct the work. Field work was also declared to the authorities responsible of the concerned marine parks. The field studies did not involve endangered or protected species.

A final continuous habitat map (scale = 1:10 000 between 0 and– 80 m and 1:25 000 beyond in the case of deeper water bodies) was realized comprising eleven habitat classes: *Cymodocea nodosa* seagrass, *Zostera marina* and *noltii*. seagrass, *Posidonia oceanica* seagrass, dead matte association, infralittoral shingle association, infralittoral soft bottoms, photophilous algae association, coralligenous assemblages, circalittoral soft bottoms, artificial habitats, offshore rocks. Ecosystem data were finally converted into presence/absence 20 x 20 m pixel layers (in order to be adapted to the pixel size related to the anthropogenic pressures, see below); the habitat corresponding to each pixel was defined by the major habitat observed within the grid (percent cover > 50%).

### Anthropogenic pressures

Drivers and pressures are here defined according to the DPSIR framework (drivers-pressures-states-impacts-responses) [45] with drivers such as the main socio-economic and socio-cultural forces increasing or mitigating pressures on the environment (rapid population expansion for example). Pressures are defined as stresses that human activities induce on the environment (e.g. wastewater), states being the condition of the environment (e.g. water quality or species richness). Impacts are defined as the effects of these pressures on the environment (e.g. biodiversity loss) and responses are what society does in order to improve the environmental situation (e.g. better wastewater treatment or regulation). Impacts may differ according to the ecosystems considered because of their variable vulnerability: all ecosystems are not threatened in the same way (functional impact, scale, frequency) and are not equally sensitive (resistance and recovery time) [46]. Here we modeled the spatial extent of anthropogenic pressures on the marine environment. We only focused on pressures that can be controlled by local stakeholders. Thus we did not take climate change issues and industrial fishing into account contrary to Micheli et al. [21] because their control appeals for high-level decisions. In addition to this, climate drivers are not considered among the MSFD’s good environmental status descriptors [47]. Ten different pressures (based on quantitative data) were considered: (1) man-made coastline (big harbours / harbours / artificial beaches, ports of refuge / pontoons, groynes, landfills and seawalls areas), (2) boat anchoring (number and size of boats observed during summer), (3) aquaculture (total area of the farms), (4) urban effluents (capacity, output), (5) industrial effluents (chemical oxygen demand), (6) urbanization (land cover), (7) agriculture (land cover), (8) coastline erosion (land cover), (9) coastal population (size and density considering the inhabitants-residents) and (10) fishing (traditional and recreational fishing areas) [see S1 Text for details]. Even if continuous pressures (e.g. wastewater) are generally distinguished from discrete pressures (e.g a groyne building), low resilience of marine ecosystems (especially *Posidonia oceanica* beds and coralligenous reefs; [48,49]) allow the combination of both pressures within the same methodology.

Data concerning the origin and intensity of these pressures are available in published databases: MEDAM [50], CORINE land cover [51], INSEE [52], MEDOBS data [53] but were also provided by Agence de l’Eau RMC and Ifremer completed with an analysis of satellite-aerial pictures and unpublished data (Andromède Océanologie). Models of the spatial extent of the pressures were built using ArcGIS 10 (ESRI) with a 20-m distance matrix. We applied a pressure curve (type *y = ae-bx*) considering the distance to the source with a negative exponential shape ranging between 100% (origin) and 0% (no more impact) to each type of pressure. We included the bathymetry to model the spread of each pressure based on literature synthesis and our expert knowledge. Details and parameters of each modeled pressure are given in S1 Text.

### Cumulative human impacts

We used a cumulative impact model following Halpern et al. [5,25] and Micheli et al. [21]. First, we assembled spatial datasets for *n* = 10 anthropogenic pressures (value *D*_*i*_) (see S1 Text) and *m* = 11 habitats (value *E*_*j*_). Secondly, all pressure layers were then log[X+1]-transformed and rescaled between 0–1 to allow direct comparisons. The sum of the different pressures per pixel was calculated. Then, cumulative impacts scores (*I*_*C*_) for each 20 x 20 m pixel were calculated according to Micheli et al. [21] and Halpern et al. [5]:

$$I_{C}=\;\sum_{i=1}^{n}\sum_{j=1}^{m}D_{i}*E_{j}*\mu_{i,j}$$

Where *D*_*i*_ is the value of an anthropogenic pressure at location *i*, *E*_*j*_ is the presence or absence of habitat *j* and *μ*_*i*,*j*_ is the impact weight of anthropogenic pressure *I* and habitat *j* [5]. Like Micheli et al. [21], values of impact weights were deduced from Halpern et al. [25].

Cumulative impact to individual ecosystems (*I*_*E*_) was calculated as follows:

$$I_{E}=\;\sum_{i=1}^{n}D_{i}*E_{j}*\mu_{i,j}$$

and impact of individual pressures across all ecosystem types (*I*_*D*_) was calculated as follows:

$$I_{D}=\;\sum_{i=1}^{m}D_{i}\text{*}E_{j}\text{*}\mu_{i,j}$$

To simplify visualization, impacts were classified in six categories depending on *I*_*C*_ values: very high (*Ic*>10); high (8<*Ic*<10); medium high (2.1<*Ic*<8); medium (0.6<*Ic*<2.1); low (0.1<*Ic*<0.6); and very low impact (*Ic*<0.1). We calculated and mapped *I*_*c*_ along the entire French Mediterranean coasts, for each marine habitat and for each water body (water bodies are here used for their interest in marine policy and as spatial references). Pixels free from any pressure (all pressures equal to null values) were not further considered.

## Results

### Marine habitats

The final continuous map of marine habitat (1:10 000 between 0 and -80 m and 1:25 000 beyond) consists of 5 785 972 pixels and covers 373 206 ha (Table 1): *Cymodocea nodosa* seagrass (506 ha), *Zostera marina* and *noltii* seagrass (572 ha), *Posidonia oceanica* seagrass (70 641 ha), dead matte association (5 693 ha), infralittoral shingle association (211 ha), infralittoral soft bottoms (102 451 ha), photophilous algae association (12 617 ha), coralligenous assemblages (2 661 ha), circalittoral soft bottoms (177 483 ha), artificial habitats (233 ha), offshore rocks (138 ha). Maps (one pixel = 10 x 10 m) are freely available on www.medtrix.fr in DONIA expert (data hosted by Medtrix are freely available for logged-in people (create account on the homepage by clicking on « register ») via the “connection” tab), see. an example of map concerning the gulf of St Tropez in S1 File). A total of 231 606 ha is considered in this study after removing 141 600 ha not concerned by any of the pressures taken into account; the removed areas are located along the deepest limits (deeper than– 100 m) of the water bodies. Most (92%) of the mapped marine bottom is covered with three habitats: infralittoral soft bottoms (38%), *P*. *oceanica* meadows (28%) and circalittoral soft bottom (25%).

**Table 1:** Analysis of the cumulative impact scores per marine habitat. Average, standard deviation (SD) and sum of the cumulative impact scores (*I*_*C*_) obtained by each 20 x 20 m cell composing each marine habitat (*j*). Areas of the habitats are indicated in ha.

Habitat	Area	Average (SD)	Sum
*Cymodocea nodosa*	446	4.85 (3.10)	54 048.88
*Zostera marina* and *noltii*	571	5.43 (1.50)	77 440.71
*Posidonia oceanica*	65 817	2.79 (2.51)	4 588 523.53
Dead matte	5 173	3.57 (2.35)	462 194.07
Infralittoral shingle association	169	5.48 (2.56)	23 153.43
Infralittoral soft bottoms	88 716	2.88 (2.35)	6 396 440.24
Photophilous algae	10 605	3.75 (3.02)	993 520.08
Coralligenous habitat	1 762	1.83 (1.83)	80 630.67
Circalittoral soft bottoms	58 049	0.73 (0.82)	1 056 582.46
Offshore rocks	21	0.85 (1.12)	435.96
Artificial habitats	109	2.56 (1.04)	6 975.16

### Anthropogenic pressures

**Fig 1:**
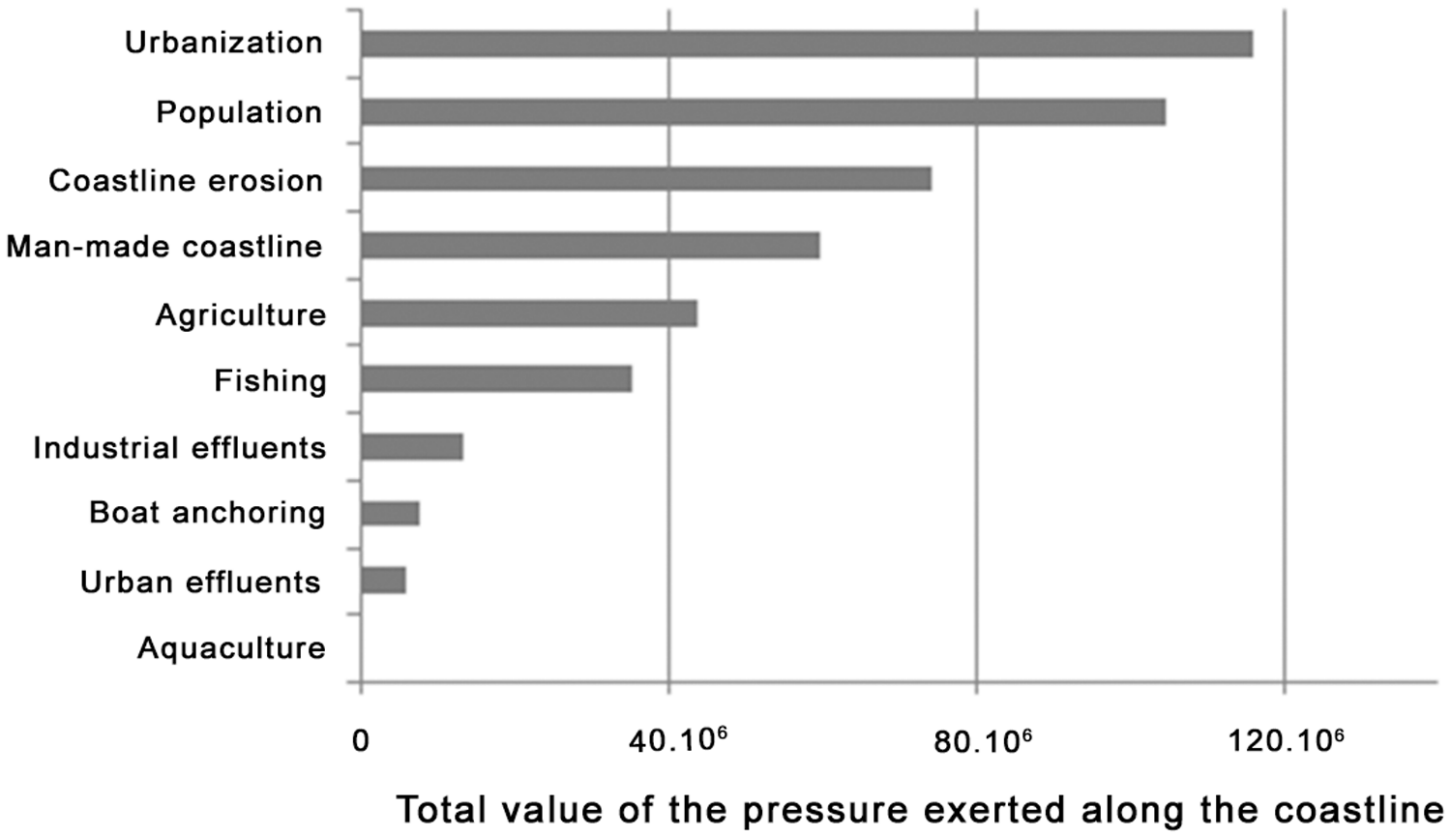
Total cumulated value (sum of all 20 x 20 m cell values concerning a pressure) of each individual pressure (no unit).

**Fig 2:**
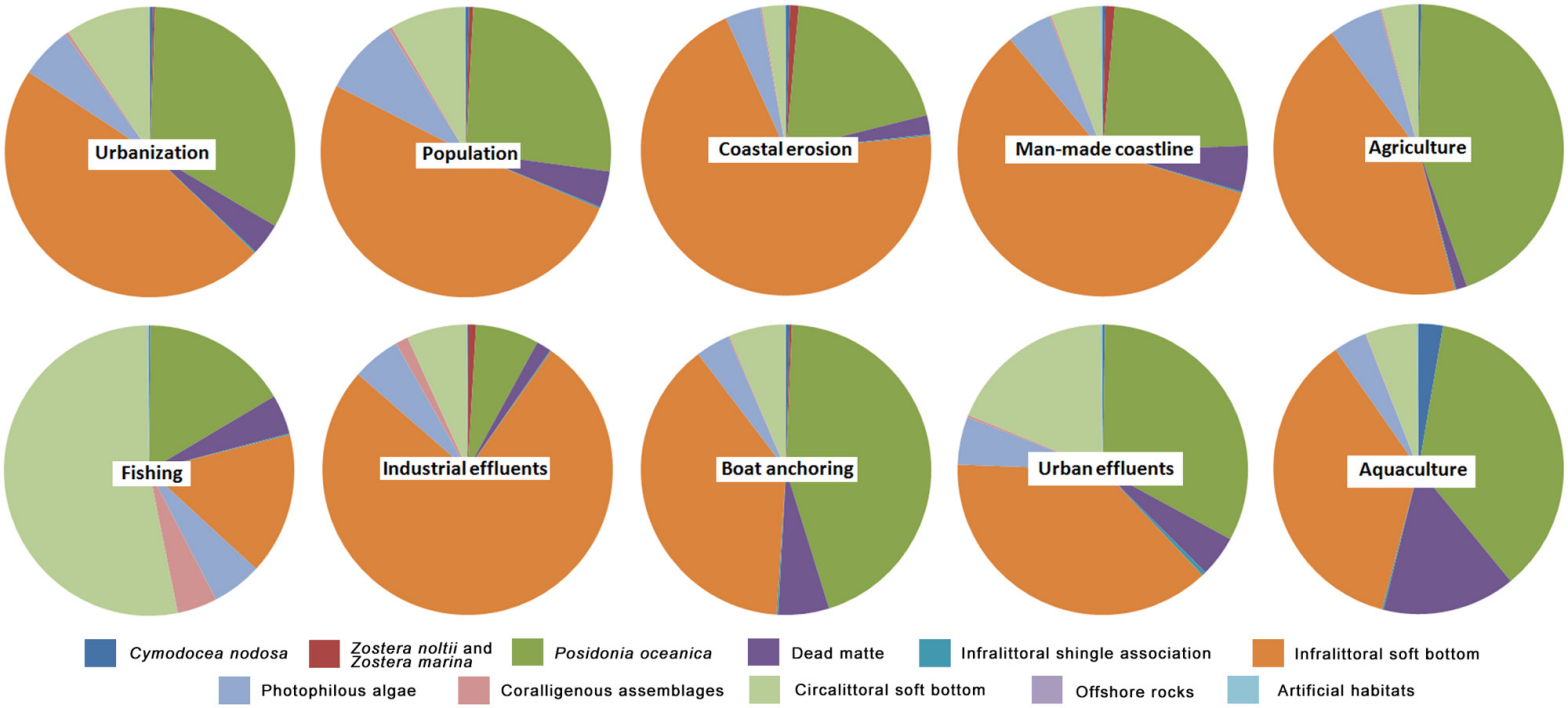
Repartition of the total cumulated value of each pressure in function of the habitats. Each pie chart indicates the repartition (in percent) of the total cumulated value (sum of all 20 x 20 m cell values concerning a pressure) of each individual pressure (name indicated in the white rectangles) in function of the habitats.

Maps concerning each of the ten pressures are available on www.medtrix.fr in IMPACT project (see box 1 and examples of maps concerning the golfe of St Tropez in S1 File). Five pressures concern more than 40% of the considered area: urbanization (70%), coastal population (54%), coastal erosion (47%), man-made coastline (43%) and agriculture (41%). Pressures showing the highest cumulated value are urbanization, coastal population and man-made coastline (Fig 1). Urbanization is the most important pressure exerted on all habitats except on coralligenous assemblages, circalittoral soft bottoms and offshore rocks where fishing prevails and on artificial habitats where man-made coastline predominates (Fig 2). All pressures affect every habitat except for *Zostera marina* and *noltii* meadows which are not impacted by aquaculture, urban effluents, agriculture and fishing, and offshore rocks which are not affected by anchoring, aquaculture and industrial effluents (Fig 2).

### Cumulative human impacts

#### Analysis per habitat

**Fig 3:**
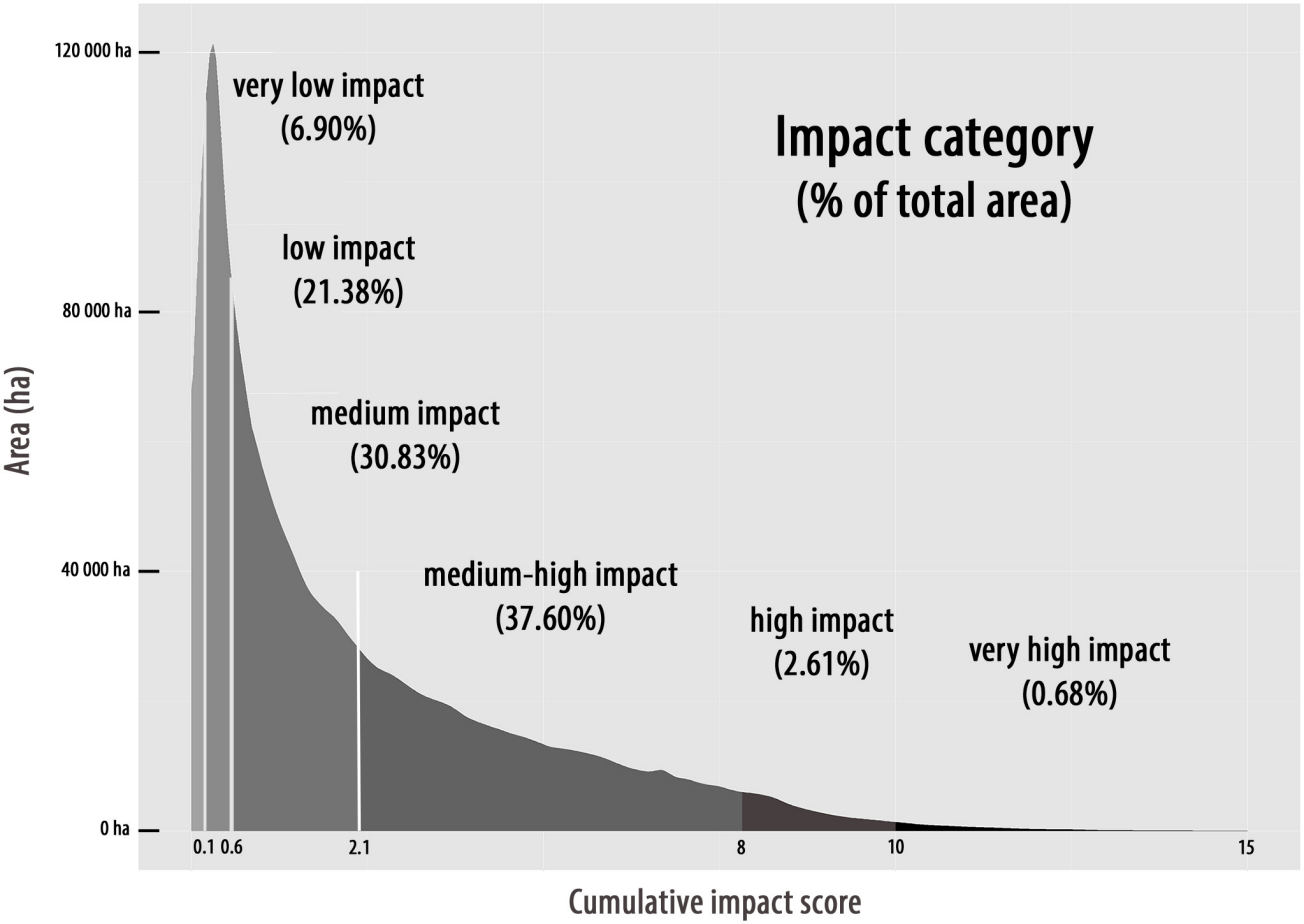
Cumulative impact scores (*I*_*C*_) depicting the area (in ha) and the percent of total area (in parentheses) that falls within each impact category.

**Fig 4:**
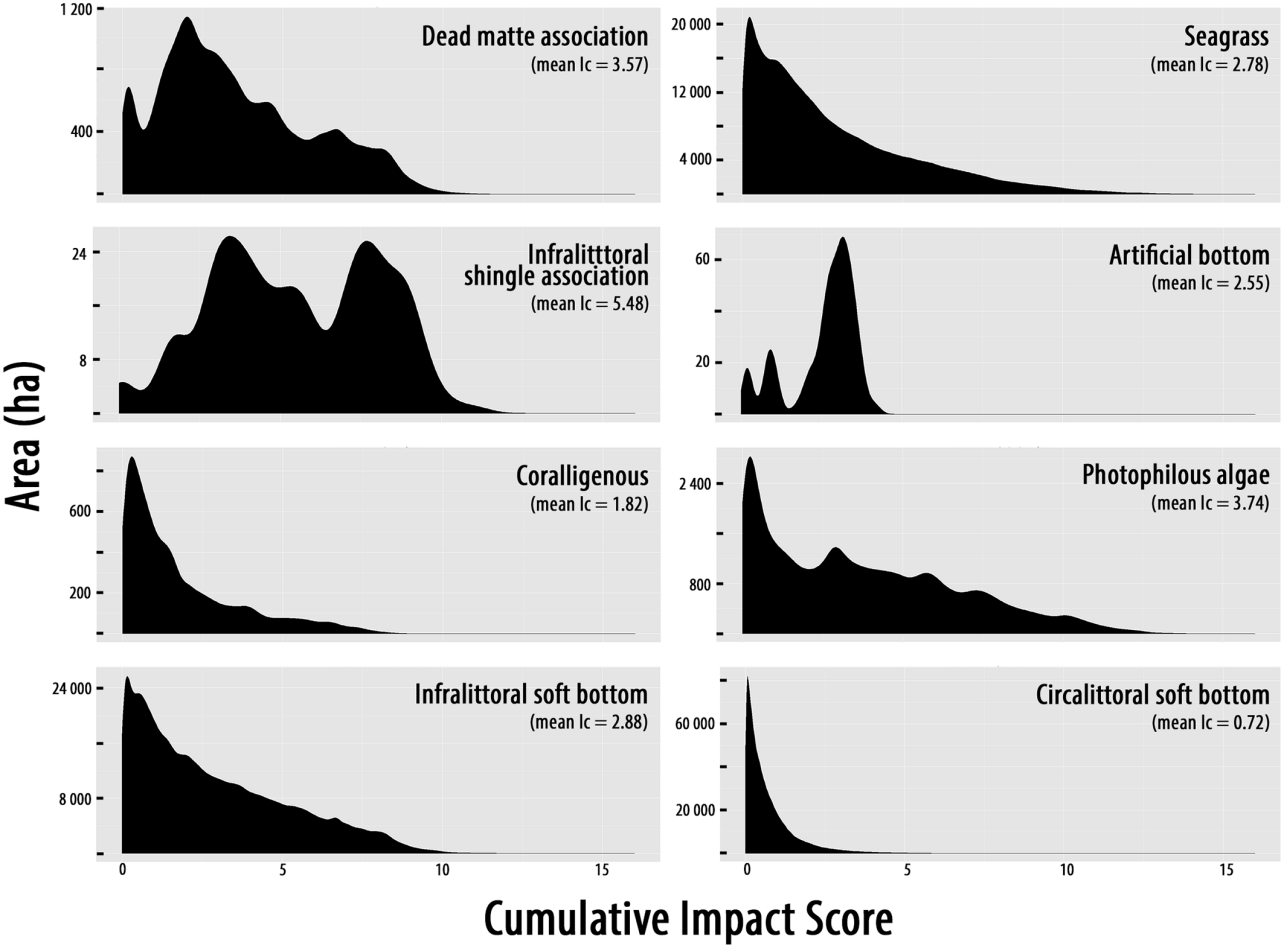
Distribution of cumulative impact scores (*I*_*C*_) for each habitat

Cumulated impact scores range between 0 and 15 (Fig 3 and S1 File). The highest sums of cumulated impact scores (*I*_*c*_) are observed on infralittoral soft bottoms and *P*. *oceanica* meadows. The strongest mean *I*_*c*_ range between 4.85 and 5.48 (medium-high impact): they are observed on infralittoral shingle association, *Zostera marina* and *noltii* and *Cymodocea nodosa* meadows. *I*_*c*_ presents the highest variance on *Cymodocea nodosa* meadows and photophilous algae (Table 1). All marine habitats are mostly subjected to medium high impacts except for coralligenous assemblages subjected to medium impacts, and circalittoral soft bottoms as well as offshore rocks concerned by low impacts (Table 2, Fig 4). Dead matte, infralittoral shingle association and artificial habitats are less subject to an *I*_*c*_ inferior to medium-high (Fig 4). Around 3.3% of habitats undergo high or very high *I*_*c*_ (especially *P*. *oceanica* meadows). On the contrary, 28.3% of habitats are associated with low or very low *Ic* especially circalittoral soft bottoms, infralittoral soft bottoms, then *P*. *oceanica* meadows (Table 2). The mean *I*_*c*_ is the highest between 0 and -15 m depth for almost all habitats (except circalittoral soft bottoms and offshore rocks absent beyond -15 m depth).

**Table 2:** Analysis of the cumulative impact categories per marine habitat. Percent of each marine habitat area affected by the different cumulative impact categories: very high impact (Ic>10); high impact (8–10); medium-high impact (2.1–8); medium impact (0.6–2.1); low impact (0.1–0.6); and very low impact (<0.1). Areas are indicated in ha.

Habitat	Area	Percent of area affected by the different cumulative impact categories					
		Very low	Low	Medium	Med-high	High	Very high
*Cymodocea nodosa*	446	4.18	5.62	8.36	61.37	12.42	8.05
*Zostera marina* and *noltii*	571	0.00	0.00	0.03	96.01	3.97	0.00
*Posidonia oceanica*	65 817	3.78	15.03	32.62	43.67	3.43	1.47
Dead matte	5 173	2.52	5.96	23.41	62.90	5.02	0.19
Infralittoral shingle association	169	1.97	1.02	7.72	68.65	19.35	1.30
Infralittoral soft bottoms	88 716	4.20	13.62	29.31	49.67	3.02	0.18
Photophilous algae	10 605	5.26	13.55	18.31	52.37	6.79	3.72
Coralligenous habitat	1 762	6.43	25.04	37.06	31.18	0.28	0.00
Circalittoral soft bottoms	58 049	15.25	43.58	34.50	6.67	0.00	0.00
Offshore rocks	21	9.94	46.39	33.92	9.75	0.00	0.00
Artificial habitats	109	0.22	7.12	16.03	76.63	0.00	0.00

#### Spatial analysis per water body

**Fig 5:**
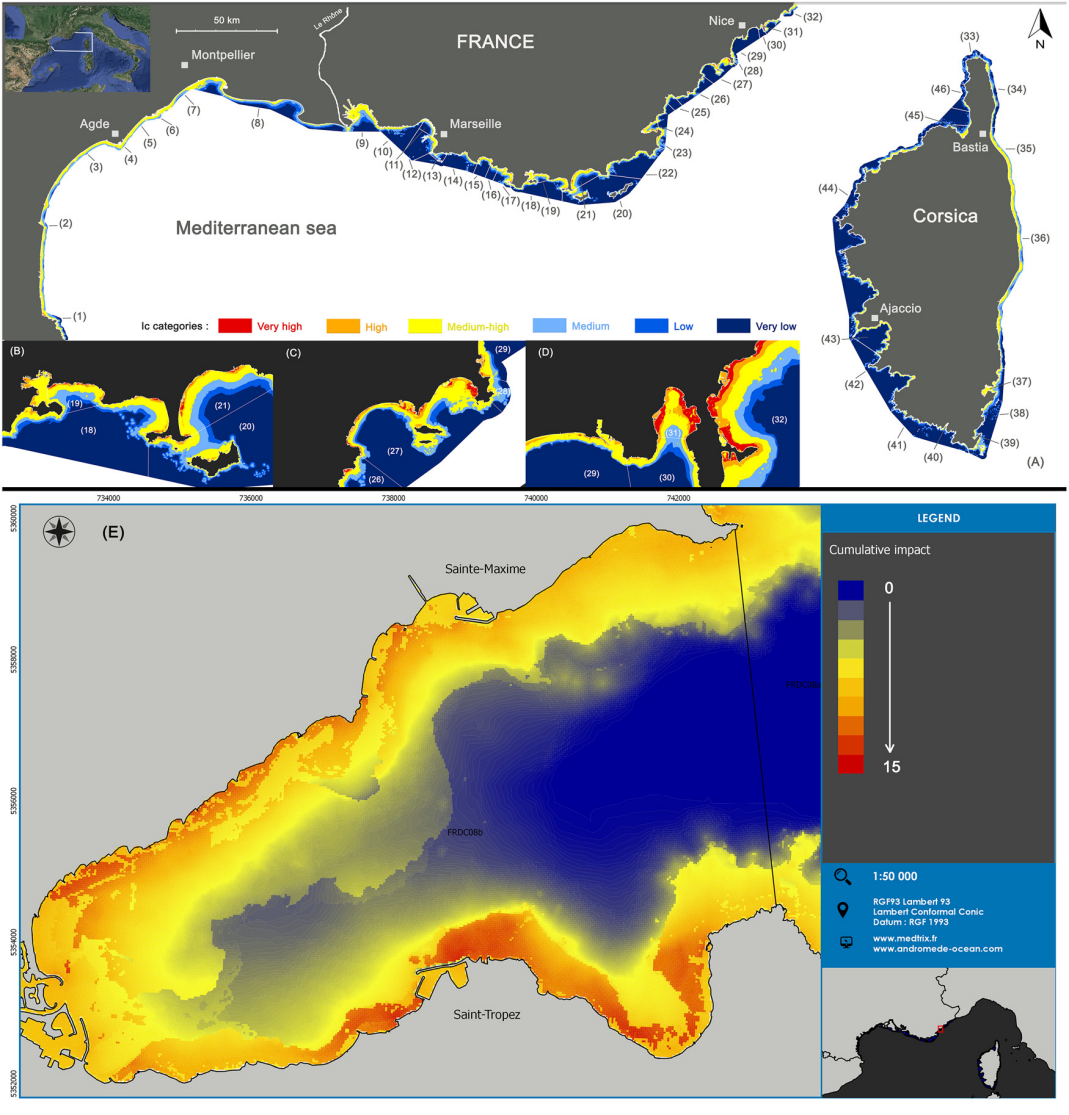
Spatial distribution of cumulative impact scores. (A) Spatial distribution of cumulative impact scores (*I*_*C*_) and localization of coastal water bodies. (B, C, D) Zooms showing how water bodies are more or less impacted (*I*_*C*_ categories). (E) Detailed map of the Golfe of St Tropez showing how the golfe is impacted (quantitative *I*_*C*_ scores) Several cities are indicated by small squares.

Concerning water bodies (localization presented in Fig 5), the highest sums of cumulated impact scores (sum of the score of each 20 x 20 m cell) are observed within water bodies 36 and 2, two of the biggest water bodies of the area (Table 3). The highest mean *I*_*c*_ correspond to medium-high impact values; they are observed among the smallest water bodies: average = 7.14 within water body 31 (also presenting the highest variance) and average = 5.82 within water body 40 (Table 3). All water bodies are mostly subjected to medium-high impacts (average ranging between 2.1 and 8, Fig 3 and Table 3). All water bodies (except two: 13 and 33 located around the Calanques of Marseille and the Northern cape of Corsica) contain areas with high or very high *I*_*c*_ (Table 3). On the contrary, all water bodies contain areas associated with low or very low *I*_*c*_ especially water bodies 8, 14, 15, 33, 34, 41, 42, 44 and 46 located in Corsica, within the Calanques of Marseille and within the Western part of the Rhône (Fig 5 and Table 3).

**Table 3:** Analysis of the pressures per water body. For each water body, the table describes its total area, its area covered by each cumulative impact score (*I*_*C*_) category (very low, low, medium, medium-high, high, very high impact), the average and standard deviation (SD) of *I*_*C*_ and the sum of the *I*_*C*_ obtained by each 20 x 20 m cell composing the water body. Coastal water bodies are numbered from West to East. Areas are indicated in ha.

Water body	Total area	Area covered by each cumulative impact category						Average *I*_*C*_ (SD)	Sum of the *I*_*C*_
		Very low	Low	Medium	Medium-high	High	Very high		
1	2 910	142	634	897	1 115	90	31	2.56 (2.53)	185 865.11
2	15 474	149	1 712	5 544	7 651	316	102	2.83 (2.22)	1 094 139.14
3	4 196	1	79	1 502	2 456	151	8	3.25 (2.10)	341 164.18
4	1 626	0	25	600	954	43	4	3.29 (2.14)	133 758.01
5	2 766	0	31	856	1 847	31	0	3.37 (1.88)	232 941.63
6	1 869	0	86	568	994	216	4	3.87 (2.71)	180 724.37
7	12 738	1 033	1 781	3 428	6 349	146	0	2.57 (2.11)	817 864.25
8	17 004	2 444	6 008	5 819	2 728	4	0	1.07 (1.23)	456 055.90
9	11 815	371	1 538	2 877	6 377	579	74	3.34 (2.63)	987 026.81
10	4 314	272	1 055	1 420	1 231	252	85	2.61 (2.91)	281 823.67
11	2 145	203	338	623	963	18	0	2.76 (2.52)	147 828.64
12	4 149	412	1 154	1 233	1 316	32	1	1.94 (2.10)	201 065.91
13	1 503	141	408	671	282	0	0	1.20 (1.07)	44 930.12
14	3 239	372	1 106	971	713	55	22	1.62 (2.15)	131 416.28
15	1 860	85	277	432	839	157	71	3.88 (3.22)	180 445.61
16	1 087	92	302	332	360	2	0	1.88 (1.88)	51 201.63
17	4 059	177	678	1 070	1 797	244	94	3.24 (2.90)	328 886.93
18	5 574	367	1 115	1 399	2 456	197	40	2.85 (2.64)	397 702.71
19	3 299	87	322	678	1 704	445	62	4.51 (3.06)	372 188.49
20	7 196	662	1 985	2 540	1 882	109	19	1.70 (1.95)	305 718.59
21	5 937	345	1 160	1 569	2 412	335	116	2.98 (2.86)	441 896.51
22	6 632	263	1 480	2 281	2 430	147	30	2.30 (2.28)	381 960.28
23	4 700	172	869	1 722	1 763	143	31	2.56 (2.46)	300 740.66
24	2 498	83	259	563	1 216	241	135	4.13 (3.24)	258 084.35
25	1 105	55	211	253	453	122	10	3.37 (3.04)	93 168.68
26	2 709	119	533	882	991	115	69	2.88 (2.90)	195 065.22
27	4 600	110	473	1 054	2 295	463	206	4.09 (3.18)	470 680.08
28	512	22	58	283	141	8	0	2.20 (2.10)	28 182.38
29	1 948	113	181	313	1 031	259	51	4.52 (3.13)	220 359.62
30	173	15	34	44	72	7	0	2.62 (2.60)	11 318.62
31	175	0	0	20	73	30	50	7.14 (3.65)	31 145.63
32	1 659	76	201	537	617	140	88	3.66 (3.30)	151 729.88
33	3 302	447	1 419	1 103	332	0	0	0.84 (1.00)	69 107.80
34	5 726	664	1 974	1 463	1 545	65	15	1.63 (1.99)	234 051.52
35	4 869	108	226	1 591	2 871	67	7	3.00 (1.95)	365 370.69
36	16 148	248	1 809	6 179	7 796	101	15	2.52 (1.87)	1 017 238.49
37	12 178	955	3 268	4 514	3 411	27	3	1.58 (1.52)	479 838.34
38	11 72	0	0	1	1 083	83	5	5.03 (1.78)	147 395.07
39	325	8	57	92	168	0	0	2.35 (1.75)	19 156.30
40	30	0	0	0	29	1	0	5.84 (1.42)	4 367.32
41	6 788	1 009	2 468	1 922	1 374	14	1	1.19 (1.45)	201 776.52
42	18 421	1 981	6 108	5 623	4 539	136	34	1.54 (1.85)	708 704.95
43	4 892	225	775	1 189	2 366	267	71	3.31 (2.80)	405 107.02
44	12 238	1 614	4 121	3 619	2 796	80	7	1.42 (1.76)	432 929.48
45	2 325	143	561	625	915	78	4	2.46 (2.46)	142 885.88
46	1 645	195	625	480	324	22	0	1.34 (1.73)	54 935.89

**Fig 6:**
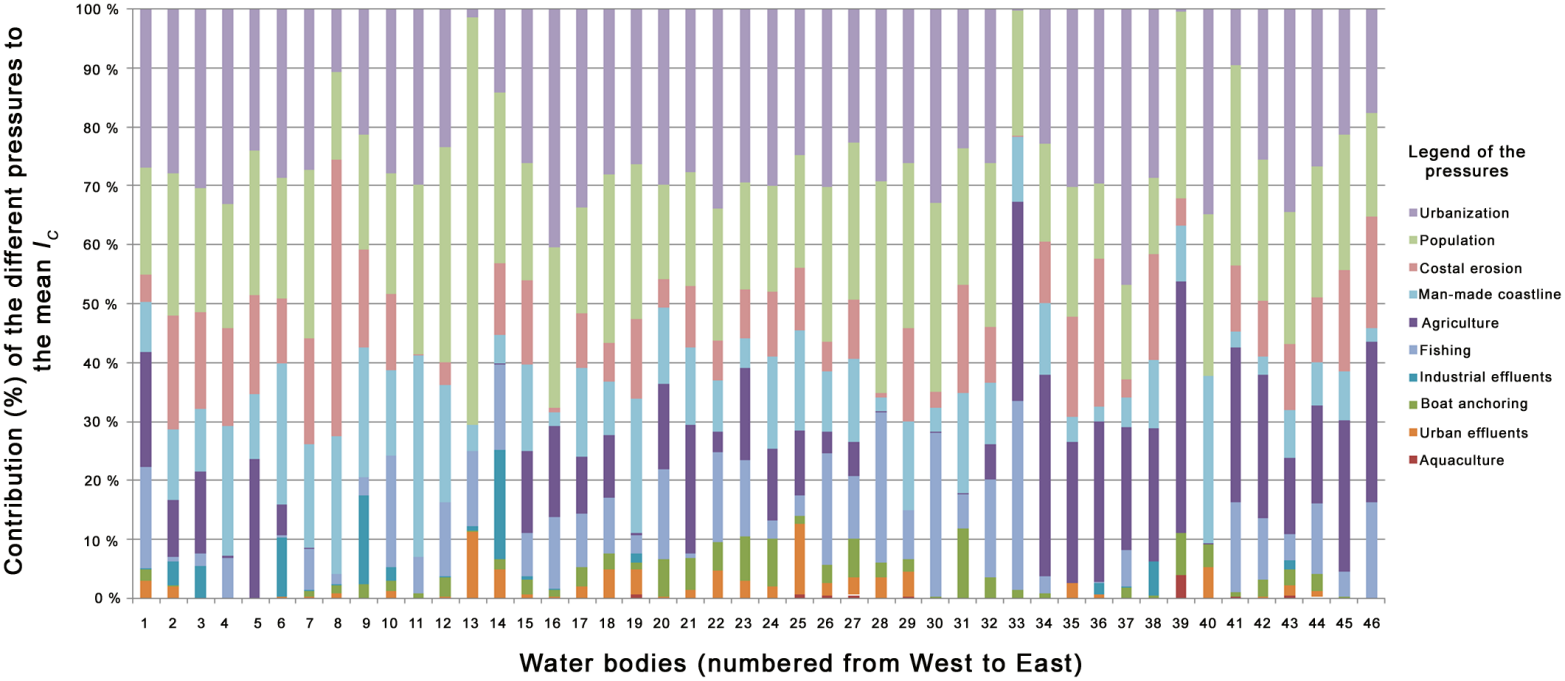
Contribution (in %) of the different pressures to the mean cumulative impact score (*I*_*C*_) of each water body. Water bodies are classified from West to East. No unit for *I*_*C*_.

Regarding the anthropogenic pressures, urbanization is the major pressure affecting all water bodies except for 5, 7, 41, 12, 13, 14, 27, 32, 38 (for which population is the major pressure); 39, 40, 33, 34 (for which agriculture is the major pressure); 9, 11 (for which man-made coastline is the major pressure); 8 (for which coastal erosion is the major pressure) (Fig 6). The relative influence of agriculture is higher for water bodies 33 to 46 (Corsica), while that of industrial effluents is higher within water bodies 9 and 14 (Fos-sur-mer and Eastern part of Marseille), and urban effluents are relatively more important between the Eastern part of Marseille and Nice (Fig 6).

## Discussion

### Fine-scale mapping of coastal habitats and pressures

This study presents the first large-scale (1700 km of coastline) continuous map of coastal Mediterranean marine habitats. It confirms the importance of areas covered by seagrass [13,54,55] and completes the coralligenous habitat distribution recently mapped [56]. Marine habitat maps are important for marine ecology and essential for managing organizations [57–59]. More importantly, the fine scale (one pixel = 20 x 20 m and even 10 m x 10 m on Medtrix) and the large area (231 606 ha) covered are available at a relevant scale for the implementation of management and conservation measures *in natura*. The initial stage of a management plan is the description of the natural components of the environment and of potential pressures and threats they are faced with. This is essential for both the identification of management priorities and the design of action plans [57,60], but also for helping managers develop dialogue with other stakeholders. Three major types of spatial information are lacking in the Mediterranean, compared to other regions such as Australia and the USA: bathymetry, habitats, and species biodiversity distribution [61]. Our results are thus important and should be completed for the rest of the Mediterranean especially for the Eastern part where information is particularly lacking.

### A heterogeneous spatial distribution of pressures

The map of cumulative human impacts highlights the widespread but heterogeneous distribution of pressures and their resulting impacts along the Mediterranean French coast. Around two thirds (68.5%) of the areas are found within medium and medium high categories, an order of magnitude similar to the one found within the French national territory waters (52.9%; [41]) and within the entire Mediterranean and Black sea (65.9% subject to medium cumulative impact [41]). Further direct comparisons with previous findings [42,62] are difficult because they have considered a larger offshore waters area and have not presented data on coastal waters. However, at several sites, visual comparison between maps produced by both studies suggests that near-coastal and offshore situations seem to be concordant and complement one another. For example, comparing to Micheli et al’s *I*_*c*_ [41] or Coll et al’s coastal-based impacts [39,42,62], the Eastern part of the Rhône shows large-scale problems with strong *I*_*c*_ in shallow (present results) and offshore waters [41], or well-preserved Northern Corsica and Camargue (Western part of the Rhône) are subjected to weak *I*_*c*_ values whatever the study and thus distance from the coast. High and very high priority areas highlighted by Giakoumi et al [42] are large and roughly include several points West of Montpellier, extend from Marseille to Nice, a small western part of the Rhône and areas at along the North Western and the South-Western coast of Corsica. Interest areas with low or very low *I*_*c*_ as defined by this present study are covered by these priority areas except two: West of Montpellier where average *I*_*c*_ is medium-high and the Northern Corsican cape where average *I*_*c*_ are the smallest of Corsica. Despite the small local differences observed (due to the finer scale and/or the greater diversity of habitats considered in the present study) the high concordance observed between the findings make the results stronger and suggest that coastal impacts keep on spreading offshore. Concerning shallow near-coastal areas that were not included in previous studies [41,63], we confirm that pressures are mostly concentrated between 0 and -15 m where the most sensitive marine habitats are also developing; seagrass meadows for example show important regressions at this bathymetric level, especially because of artificial coastlines [64,65].

The most important pressures (considering both area and intensity) are urbanization, coastal population, coastal erosion and man-made coastline, which are directly related to coastal developments and territorial planning. Our results might help stakeholders prioritize their policy actions. Two regions should particularly draw attention: the bay of Villefranche-sur-mer (close to Nice) and Bonifacio (Southern cape of Corsica), respectively water bodies 31 and 40 presenting the highest mean *I*_*c*_ and variance. Urbanization is broadly the major pressure exerted but its impact depends on the sites because of the variety of the surrounding seabed nature (and thus vulnerability). For instance, the weight of the impact is lower around a largely urbanized site surrounded by soft bottoms than when by more vulnerable seagrass meadows. Where urbanization is not the major pressure, town-planner’s attention should be drawn to the impact of man-made coastline that is particularly important, such as around the harbour of Marseille and its neighbour Fos-sur-mer (two major industrial and commercial harbour areas), but also on coastal erosion which occurs around the largest river, the Rhône. Similarly, coastal population is the major pressure affecting several areas known for their touristic attractiveness, although the seasonal population was not taken into account (the offshore bar between Agde and Sète, Palavas-les-Flots, Marseille, Frioul island, Cassis, Cannes, Porto Vecchio) and where urbanization remains paradoxically quite contained. These areas should be particularly kept under surveillance because urbanization is likely to increase there. Finally, agriculture mostly affects Corsican coasts, the last region where farmlands still remain in coastal areas. Corsica is actually a well-preserved island with the lowest population density of Metropolitan France (36.3 inhabitants/km²—INSEE data) and a wild coast appreciated by tourists. Nevertheless, Corsica had the highest French demographic growth since 2006 (1.3% / year compared to 0.6%/year at national level—INSEE data) especially around the two coastal cities of Ajaccio and Bastia. Corsica’s coastal population pressure should thus particularly be monitored.

Our results show that two areas are particularly preserved from the analyzed anthropogenic pressures and should thus deserve particular attention and protection in the future: the Calanques of Marseille and the Northern cape of Corsica. Moreover, these regions present medium to high levels of biodiversities [39,40,66] and aesthetics [67]. The strong interest of these sites is indeed taken into account as a national marine park was created in the Calanques of Marseille in 2012 [68] and a project is under consideration within the Northern cape of Corsica since summer 2014 [69].

### Threatened habitats

Because of the methodology, high *I*_*c*_ are associated with numerous pressures. Almost all habitats are affected by all pressures except the closest or the furthest from the coast (*Zostera marina* and *noltii* meadows, offshore rocks). This highlights the importance of coordination for action plans focusing on pressures threatening coastal marine habitats.

Principally derived from other damaged habitats, a relatively weak proportion of dead matte and artificial habitats are logically subjected to low cumulative impacts in comparison with other habitats. Indeed, dead matte is the biological remains of dead *P*. *oceanica* meadows and artificial habitat is a man-made habitat replacing natural ones. However, these habitats should not be abandoned because they are interesting substrates for restoration measures. For example, seedlings of *P*. *oceanica* transplanted on dead matte show a higher survival rate than on sand or shingle substrate [70,71]. Artificial structures may also be directly (artificial reefs, green infrastructures), or indirectly (i.e. colonization of pipelines) used for biodiversity management. For instance, several ongoing projects aiming at using artificial habitats (harbours, seawalls, groynes) to boost biodiversity might be more successful if local pressures are not too high [72,73].

Infralittoral shingle association is also largely submitted to medium and high cumulative impacts but for other reasons: it covers a relatively small area and it is localized at shallow depths (shingles carried by rivers are localized near the coastline) and where pressures are strong (major cities are located along the rocky coast). Finally, the most important cumulative pressures occur within the largest habitat, which is also the most sensitive: seagrass meadows. Seagrass meadows have to be a priority for management and conservation plans, being among the most efficient ecosystems considering the ecosystem services provided per surface unit [74]. Despite different protection measures *P*. *oceanica* meadows already beneficiate from (European Habitats Directive, Barcelona Convention, Bern Convention), they are still strongly damaged [64,75,76].

The deepest habitats (coralligenous habitat, circalittoral soft bottoms and offshore rocks) are relatively less subjected to pressures (among the ones considered for this study) than the other habitats because they are generally more distant from the coast. Although many pressures are known to impact these habitats [27,77], we show that they are mostly threatened by fishing in the present study. Even non-industrial fishing practices (traditional, recreational, spearfishing) can cause rapid and substantial negative effects as well as represent an important part of the total fish catches (30% for example on the French Atlantic coasts) [78,79]. Despite the importance of this pressure, particularly on coralligenous habitats, very little actions have been taken to limit this recognized threat (medium *I*_*c*_) [49] while outputs could be controlled and size or catch of fish limited.

### Utility for management despite several limitations

According to Giakoumi et al [42], “a prerequisite to quantification of threats and effective implementation of conservation actions is the acquirement of fine scale spatial data”. Maps of marine habitats and of pressures that can impact these habitats are the basic knowledge necessary for management work (see the first paragraph of discussion). Developing and measuring indicators of water quality or of ecological status of habitats, also needs to locate and assess the pressures acting locally; these are generally roughly estimated [80–82] and our maps of pressures and cumulative impacts might help to refine these works. Knowing precisely where are sensitive habitats and how they are impacted is essential before the deployment of adequate mitigation measures [42]. Our work will also make easier the measurement of management action efficiency; for example once anchoring is targeted as the major impacting pressure in an area, managers can choose how and where they want to contend with: mooring prohibition, mooring buoys, access to maps for boaters (i.e. Donia application [44]). Our work may help stakeholder to prioritize their means: protect areas where cumulative impacts are low or very low or try to act on “controllable” pressures where cumulative impacts are medium to high. Finally, these maps are like photos of the state of the coast at a *t* time and might be done again in five to six years in order to see the eventual changes, compare similar sites and test the efficiency of different management choices.

Lastly, when interpreting the results, it is important to consider the data limitations and uncertainties inherent in this work. First, we assumed a linear relationship between pressure intensity and impact on habitat and ignored thus the existence of thresholds that certainly do exist. Like previous studies [41,42,62], we skipped the thresholds because there is a lack of information about them. Similarly, for the same reasons our analyses did not include eventual synergy or antagonism between pressures acting at the same place. Secondly, maps represent what we know in 2014; it is thus possible that some pressure or habitat is invisible on the maps because the information was unavailable at this time. Moreover, available data were collected during an extended period of time (four years) so they represent an average situation (besides without any seasonal variation) even if local managers easily communicate us their feed-back on the maps now available on line. Then detailed information are hard to access and thus data could be refined if they became available: use a finer grid size (presently 20 x 20 m) for pressures and avoid to degrade the presently available information for habitats (1:10 000 map), use more precise denominations for habitats (i. e. levels within habitat types, plant densities for *P*. *oceanica*, data on species assemblages). Similarly, in perspective, numerous new pressures may be added if the data (raw data or model outputs) are available: climate change, alien species, industrial fishing (including trawling), diving activities or marine traffic for example.

## Conclusion

Our study provides the first maps of habitat and cumulative pressures distribution on the French Mediterranean coastline. These maps are now urgently needed for marine systems which are deteriorating faster than other ecosystems [2]. They will be very relevant to biodiversity conservation, to help communicate, prioritize environmental issues, make political choices, better understand the links between anthropogenic pressures and ecological status of coastal systems, and to apply and coordinate management measures. Combined with maps of species distribution they may be viewed as maps of potential biodiversity loss and will help evaluate the objectives of the European directives (MSFD, 2008/56/EC and WFD, 2000/60/EC). The maps we have produced here represent a single snap shot of marine habitats and pressures but they could easily be updated and used to fuel models to predict future impacts if appropriate scenarios are available [30]. On the long term, mixing these kind of extensive spatial mapping of habitats and pressures with ecological modeling will prove particularly useful because “long-term and large-area ecological processes are particularly poorly understood; and yet, in a number of areas, issues and well-defined policies have not been sufficiently developed” [2]. In particular, field data concerning ecosystem responses to pressures and thus the relationship between cumulative impact scores and ecosystem condition should now be considered a top priority [41].

## Supporting Information

S1 Fig Origin of the data used for marine habitat mapping on the Western part of the French Mediterranean coast (Languedoc-Roussillon French region). The study considers the coastline included within the water bodies. We particularly focus on the shallow part: between 0 and -80 m. After a bibliographic synthesis, we gathered and homogenized data on habitat maps. Gaps were completed with the program DONIA with a fine scale (1:10 000 map) between 0 and -80 m and a lower resolution (1:25 000) beyond.
(TIF)

S2 Fig Origin of the data used for marine habitat mapping on the Eastern part of the French Mediterranean coast (Provence Alpes Côte d’Azur French region). The study considers the coastline included within the water bodies. We particularly focus on the shallow part: between 0 and -80 m. After a bibliographic synthesis, we gathered and homogenized data on habitat maps. Gaps were completed with the program DONIA with a fine scale (1:10 000 map) between 0 and -80 m and a lower resolution (1:25 000) beyond.
(TIF)

S3 Fig Origin of the data used for marine habitat mapping on the Eastern part of the French Mediterranean coast (Corsica French region). The study considers the coastline included within the water bodies. We particularly focus on the shallow part: between 0 and -80 m. After a bibliographic synthesis, we gathered and homogenized data on habitat maps. Gaps were completed with the program DONIA with a fine scale (1:10 000 map) between 0 and -80 m and a lower resolution (1:25 000) beyond.
(TIF)

S1 Text Details and parameters of each anthropogenic pressure. (DOCX)

S1 File Examples of detailed maps obtained during the study for the different anthropogenic pressures, marine habitats, cumulative impact scores (*I*_*C*_) and cumulative impact categories. The Gulf of St Tropez is taken as an example. All the detailed maps are available: www.medtrix.
(PDF)
