# Supplementary figures and images for: Fine-Scale Cartography of Human Impacts along French Mediterranean Coasts: A Relevant Map for the Management of Marine Ecosystems
Source: PLoS One. 2015 Aug 12;10(8):e0135473. doi: 10.1371/journal.pone.0135473 (PMC4534390; doi:10.1371/journal.pone.0135473)

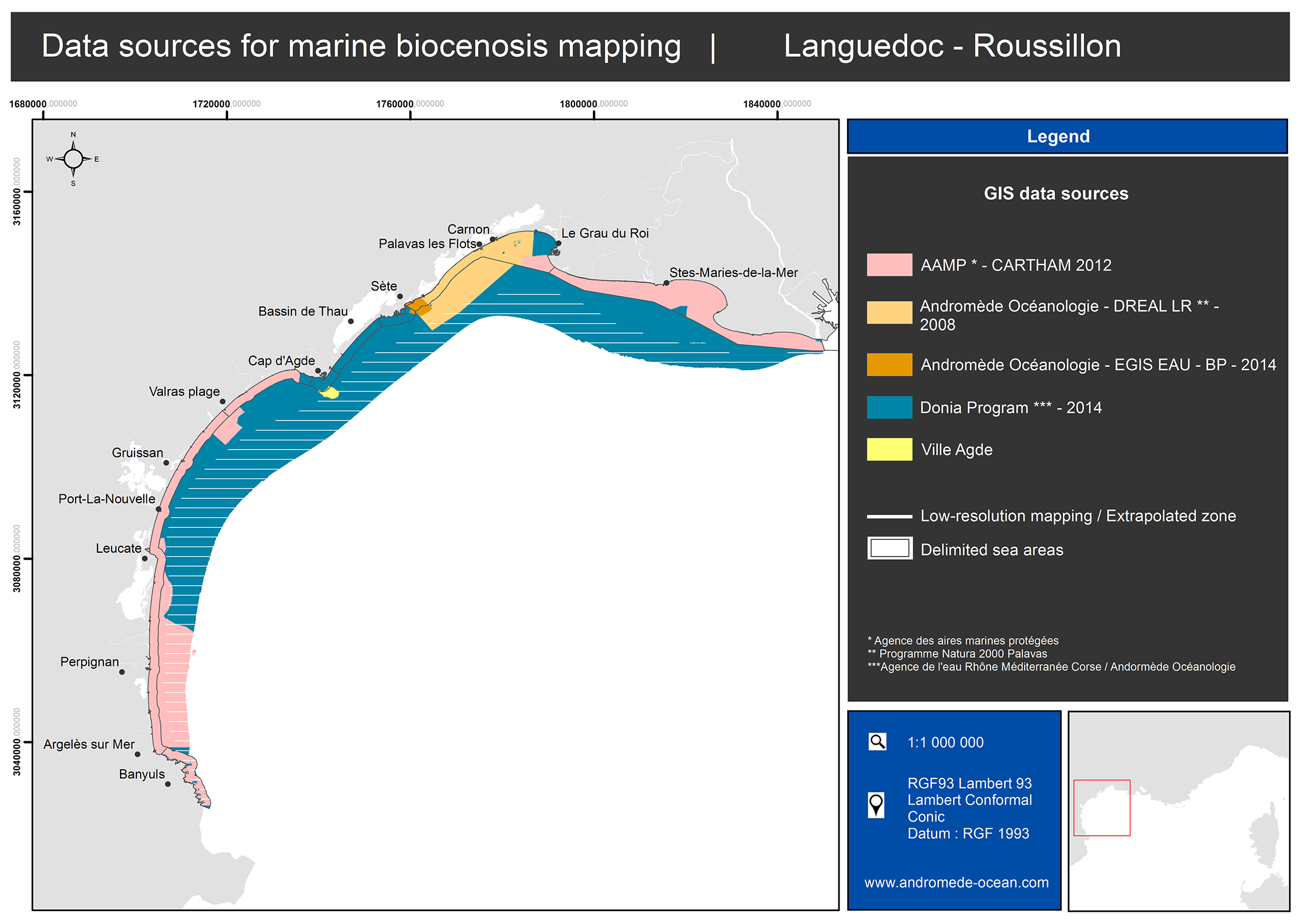

Supplement: S1 Fig — The study considers the coastline included within the water bodies. We particularly focus on the shallow part: between 0 and -80 m. After a bibliographic synthesis, we gathered and homogenized data on habitat maps. Gaps were completed with the program DONIA with a fine scale (1:10 000 map) between 0 and -80 m and a lower resolution (1:25 000) beyond. (TIF) [file pone.0135473.s001.tif]

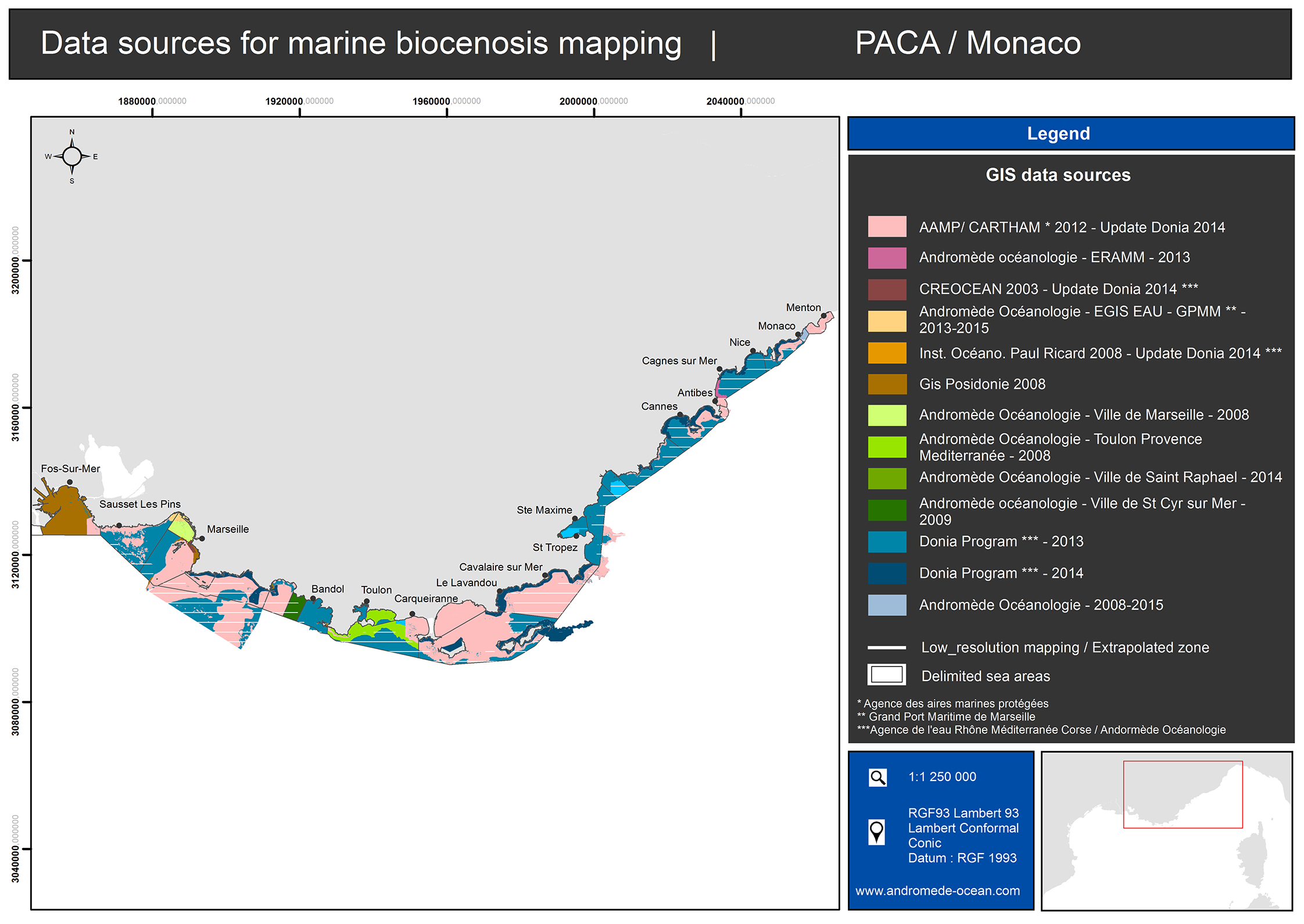

Supplement: S2 Fig — The study considers the coastline included within the water bodies. We particularly focus on the shallow part: between 0 and -80 m. After a bibliographic synthesis, we gathered and homogenized data on habitat maps. Gaps were completed with the program DONIA with a fine scale (1:10 000 map) between 0 and -80 m and a lower resolution (1:25 000) beyond. (TIF) [file pone.0135473.s002.tif]

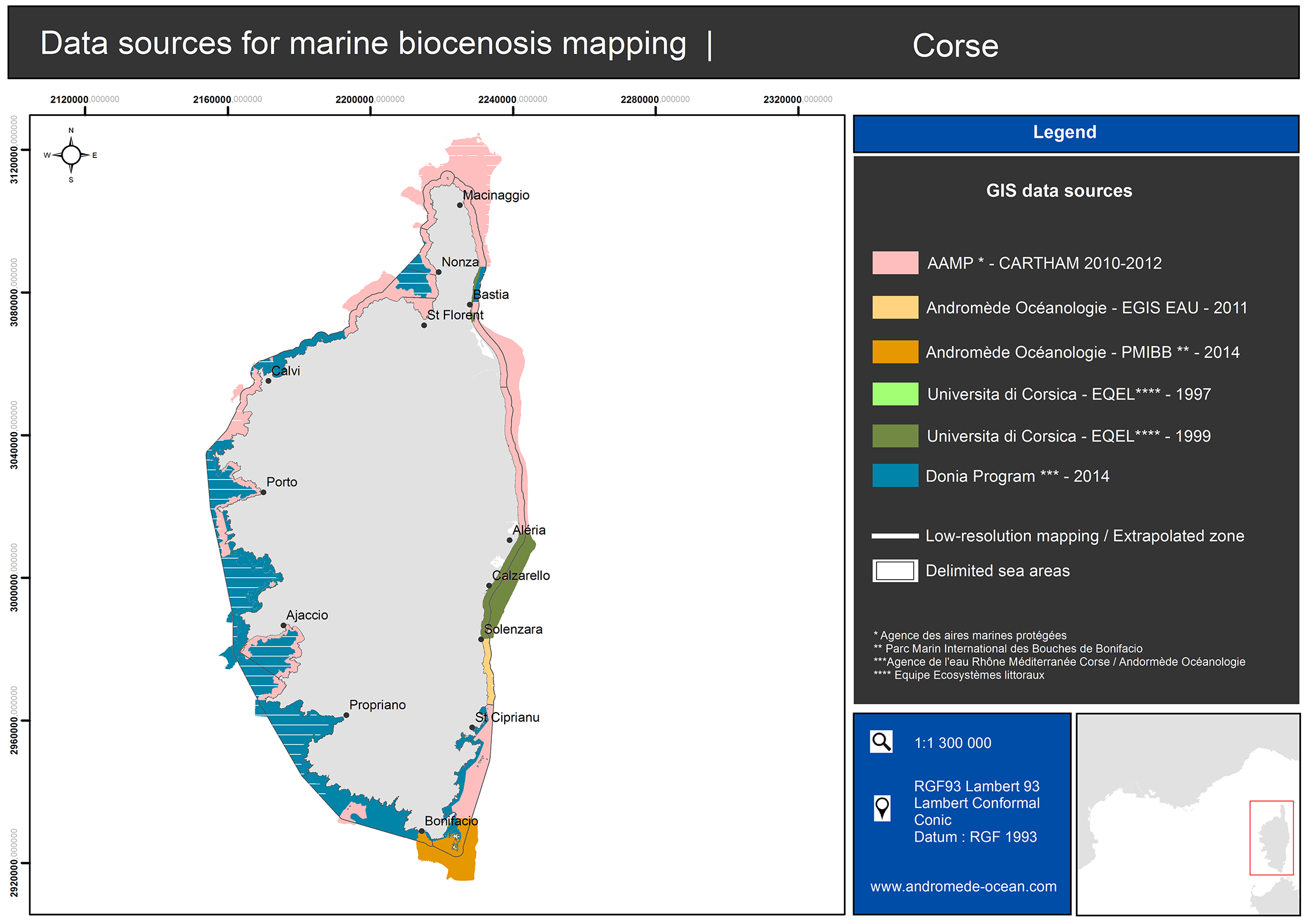

Supplement: S3 Fig — The study considers the coastline included within the water bodies. We particularly focus on the shallow part: between 0 and -80 m. After a bibliographic synthesis, we gathered and homogenized data on habitat maps. Gaps were completed with the program DONIA with a fine scale (1:10 000 map) between 0 and -80 m and a lower resolution (1:25 000) beyond. (TIF) [file pone.0135473.s003.tif]
