# Supplementary material for: Fine-Scale Cartography of Human Impacts along French Mediterranean Coasts: A Relevant Map for the Management of Marine Ecosystems
Source: PLoS One. 2015 Aug 12;10(8):e0135473. doi: 10.1371/journal.pone.0135473 (PMC4534390; doi:10.1371/journal.pone.0135473)
Supplement: S1 Text — (DOCX) [file pone.0135473.s004.docx]

**Supplementary Online Materials**

**Methods**

**General Approach**

We used a 4-step process cumulative impact model, an approach proposed by Halpern et al [1] and used then by Selkoe et al and Micheli et al [2,3]. First, spatial data were pooled for each anthropogenic pressure (*Di*) and each habitat (*Ej*). Second, a single and unitless scale was used for the pressures by log[X+1]-transforming and rescaling them between 0-1, and habitat data were converted into 20 x 20 m presence/absence layers. Third, pressure-by-habitat combinations (pressure layer multiplied with each habitat layer) were created for each 20 x 20 m marine cell and then multiplied by the appropriate vulnerability weighting variable (*uij*) (= relative impact of a pressure on an habitat) calculated on the basis of an expert survey [4]. The sum of these weighted pressure-by-habitat combinations represents the relative cumulative impact of human activities on all habitats in a particular 20 x 20 m cell. Finally, to make easier the visual display of results, according to Micheli et al and Halpern et al [3,5], impacts undergone by marine habitats were classified in six ecologically meaningful categories for the Mediterranean sea and inspired by[1] on the basis of I*_C_* values: very high (Ic>10); high (8-10); medium high (2.1-8); medium (0.6-2.1); low (0.1-0.6); and very low impact (<0.1).

This study focuses on Mediterranean French coastal waters. A total of 10 pressures (Table S1) and 11 marine habitats spatial datasets were pooled and used. These pressures are assumed to capture different impacts endured by the coastal habitats. Maps can be viewed at [www.medtrix.fr](http://www.medtrix.fr) (“DONIA expert” project for the habitats and “IMPACT” project for the pressures and I_c_). We describe below the data layers used in the analyses. The pressures considered and their data source and resolution are summarized in the table S1.

Table S1. List and characteristics of spatial data layers representing pressures influencing marine ecosystems used to assess and map cumulative human impacts along the Mediterranean coastline.

| Pressures | Resolution | Source |
| --- | --- | --- |
| Coastal engineering | 20 m | Meinesz, Chancollon, Garcia, & Markovic, 2010  MEDAM on www.medam.org |
| Boat anchoring | 20 m | MEDOBS on [www.medtrix.fr](http://www.medtrix.fr)  Satellite / aerial pictures |
| Aquaculture | 20 m | 2013 data base provided by French Water Agency and Ifremer  Satellite / aerial pictures |
| Urban effluents | 20 m | 2013 data base provided by French Water Agency and Ifremer  Outputs and capacity of discards on http://assainissement.developpement-durable.gouv.fr/ |
| Industrial effluents | 20 m | 2013 data base provided by French Water Agency and Ifremer |
| Urbanization (land cover) | 20 m | CORINE Land Cover |
| Agriculture (land cover) | 20 m | CORINE Land Cover |
| Coastline erosion | 20 m | CORINE Land Cover |
| Costal population (size and density) | 20 m | INSEE |
| Fishing (traditional and recreational) | 20 m | MEDOBS on [www.medtrix.fr](http://www.medtrix.fr) + unpublished field data between 2008 and 2014 |

**Coastal engineering: harbours, artificial beaches and other reclamations from the sea**

Coastal engineering cause modifications in initial conditions and disrupt local hydrodynamics and siltation [6]. Actually, costal engineering are reclamations from the sea (harbours, groins, man-made beaches,…) causing irreversible destructions of sublittoral seabed by covering and leading important changes in the hydrodynamics around these layouts [6]. The inventory of the reclamations from the sea along the French Mediterranean coast [7] showed 11.10 % of man-made coastline including 3.03 % irreversibly destroyed shallow (0-20 m) bottoms (http://www.medam.org). The impact distance is hard to estimate but studies concerning *Posidonia oceanica* seagrass beds, the most sensitive marine ecosystem in shallow Mediterranean waters, may help [8,9]. The ecological status of this ecosystem declines as the driver source comes closer, with an impact that may be visible along several kilometers [10]. A recent large-scale (800 km) study estimates impact distances from the spatial changes occurred between historical (1922) and present aerial pictures considering coastal development (layouts identified by the MEDAM database) regrouped in four categories. Our models use these mean maximal impact distances: (1) 15 km for very large harbours and installations (Marseille and Toulon) (2) 10 km for harbours (3) 3 km for ports of refuge, artificial beaches, (4) 1 km for pontoons, groins and landfills. Driver sources come from the MEDAM GIS layer (http://www.medam.org). A 20-m distance matrix is created. A negative exponential pressure curve is applied starting from the sources and decreasing between 100 and 0 % with the following equations: (1) y=99.175e-0.304 for big harbours; (2) y=99.175e-0.456x for smaller harbours; (3) y=99.175e-0.912x for artificial beaches and ports of refuge and (4) y=99.175e-0.912x for pontoons, groins and landfills. Bathymetry is taken into account with a theoretical loss of 10 % per 10-m depth group.

**Anchoring**

Boat anchoring (free mooring) may cause important damages to shallow ecosystems especially seagrass meadows [11] on which the impacts are more visible and studied. Anchors and their chains mechanically destroy the meadows in particular at their deeper limits where meadows are less dense and more sensitive [12]. *Posidonia oceanica*, the most common seagrass in Mediterranean sea is also the species that presents the lowest growth rate (1-10 cm per year) in the world [13,14]: it is thus particularly little resilient, making any damage irreversible at a human scale [8].

Anchorage GIS layer was created on the basis of MEDOBS data available at [www.medtrix.fr](http://www.medtrix.fr) (anchoring boats counted from a plane during summers, see more details on <http://www.observatoire-mer.fr/en>). Anchorage areas and the mean number of boats (with their size < or > 20 m long) observed during summer are completed with satellite and aerial [15] <http://www.bing.com/maps/>) pictures, and [www.sea-seek.com](http://www.sea-seek.com). Each anchorage area is classified among one of three categories depending on the mean number of boats: 1) <100 boats, 2) > 100 and < 200 boats and 3) > 200 boats. An increase to the upper category is applied in areas counting big vessels (> 20 m long). A 20-m distance matrix is created. A one-kilometer buffer zone is created around the origins of sources with an exponentially decreasing impact depending on the categories and the following equations: 1) *y=41.084e-3.776x*, 2) *y=69.235e-4.079x*, 3) *y=99.175e-4.56x.* Bathymetry is taken into account by applying an increasing impact (*y=-0.004x3 + 0.3624x2 – 6.5008x + 22.681)* between 0 and – 5 m, a maximal impact between – 5 and -15 m (no buffer) and a decreasing impact between -15 and – 45 m with a theoretical loss of 10 % per 10-m depth group.

**Aquaculture**

Aquatic farms are used for the production of fish, mollusks, shellfish and aquatic plants. Food supply, high density of organisms and materials used may cause damages to the marine environment. IUCN [16] classifies the local potential negative impacts within five domains: introduced marine species, organic matters in the effluents, pathogen transfer, therapeutics and other products, genetic degradation of wild populations. The environmental impact of aquatic farms is the subject of numerous studies [17,18]. They show that an excess in organic matters and nutrients decreases the available luminosity by eutrophication. The degradation of these components leads then to the formation of sulphides causing a sedimentary hypoxia with a toxic effect on seagrass [19]. This negative effect is maintained even after the cessation of activity because of the destabilization and the accumulation of organic matters within the sediment [20]. The enrichment in organic matters is the most important just under the farms and may be detectable until 50 [17] or 84 m around [21]. An impact on seagrass meadows (especially on the vertical growth) is observed until 800 m around [22], and some authors recommend a minimal distance of 400 m between farms and meadows [19]. Nevertheless, an anormal increase in epiphytes is observed on seagrass even at 3 km of fish farms, but without any significant alteration of the meadows [23]. As for the benthic macrofauna, an impact is noted until a distance of 907 m but this distance strongly varies according to the sediment nature (rough or sludgy) with a tendency to be less detectable on rough soft bottoms preferred by seagrass [24].

Aquaculture GIS layer is created on the basis of a database provided in 2013 by the French Water Agency and Ifremer and completed with a research of new companies on the directory and of new sites on aerial pictures [15]. Each aquatic farm is classified within a category in function of its cover (< or > 3977 m²) after the drawing of polygons on the map. The limit area is established using a Jenks natural breaks classification method. A 20-m distance matrix is created. An exponentially decreasing driver is modeled until 500 m for the smallest farms with the following equation *y=99.175e-9.119x* and until 1000 m for bigger farms with the following equation: *y=99.175e-4.56x.* The influence of the bathymetry is considered from 0 to – 100 m according to a theoretical loss of 10 % per 10-m depth group.

**Urban effluents**

Urban effluents are generally given off *via* a pipe within a marine area characterized by a strong hydrodynamics able to guarantee their dilution. It results in a highly variable spatial influence of the effluents depending on the dilution and spread of contaminants by waves and currents [25]. These effluents have clear negative impacts on marine habitats particularly on *Posidonia oceanica* meadows [26]. The influence of effluents on the benthic macrofauna and on habitats was studied at different distances from the discard pipe et for different types of discards in the framework of annual surveys [27–31] and the modeling of plumes is sometimes realized [28,32,33]. A work comparing the areas covered by *P. oceanica* meadows before and after 90 years (1920-2010) of coastal development along a French region evidenced the importance and the localization of regressions in link with the building and the discarding of different pipes [34]. Another study has showed that seagrass beds are contaminated by heavy metals until at least 5 km from the driver source like at Canari in Corsica (France) [35]. Concerning the soft bottoms, a discard might influence the benthic assemblages until 8 km according to a negative exponential curve starting at the driver origin [36].

The GIS layer regrouping all waste water discard points is built on the basis of a database provided in 2013 by the French Water Agency and Ifremer. This is completed and corrected in function of different data sources: the pipes localized on 1:25 000 IGN maps, discards localized on the platform eau France ([www.eaufrance.fr](http://www.eaufrance.fr)) and our field knowledge. The outputs (in m^3^/day) and the capacity (population equivalent) of each discard are obtained from http://assainissement.developpement-durable.gouv.fr/. Discards are classified into five categories according to their output: 0) ≤ 1 500, 1) ]1 500; 5 000], 2) ]5 000 ; 10 000], 3) ]10 000; 50 000], 4) > 50 000 m^3^/day; and their capacity in population equivalent: 0) < 4 000, 1) ]4 000; 10 000], 2) ]10 000; 40 000], 3) ]40 000; 100 000] and 4) < 100 000 population equivalent. Categories were chosen on the basis of the output and capacity distribution. A maximal impact distance is then given according to the mean note mixing the notes concerning capacity and output: 1 km for a note of 0 or 1 (a), 3 km for a note of 2 (b), 5 km for a note of 3 (c) and 10 km for a note of 4 (d). A 20-m distance matrix is created. An exponentially decreasing driver is modeled according to the following equations in function of the mean note obtain: *y=99.175e-4.56x* (note=a), *y=99.175e-1.52x* (note=b), *y=99.175e-0.912x* (note=c), *y=99.175e-0.456x* (note=d). Hydrodynamics is considered by subtracting the force vector of the current per pixel (obtained from the 2010-2011-2012 Previmer model (www.previmer.org)). Bathymetry (from SHOM) is considered with a theoretical loss of 10 % per 10-m depth group.

**Industrial effluents**

Industrial growth is often accompanied by an increase of contaminant discharge within the natural environment [37]. These may strongly impact entire ecosystems [38]. There is no information concerning contaminant fluxes to the sea at a large homogeneous scale within Mediterranean. In this absence, one of the most commonly used indicators of water quality is the COD (Chemical oxygen demand), an indirect measure of the amount of organic compounds in water. The GIS layer regrouping all the industrial discards within the sea and their COD value is built on the basis of a database made available by the French Water Agency and Ifremer in 2013. A 20-m distance matrix is created. Industrial effluents are assumed to exponentially decrease from their origin until 5 km for COD < 100 mg/l according to *y=99.175e-0.912x*, until 10 km according to y=99.175e-0.456x for COD ranging between 100 and 1000 mg/l, and until 20 km according to *y=99.175e-0.228x* (for COD > 1000 mg/l. Bathymetry (from SHOM) is considered in each model with a theoretical loss of 10 % per 10-m depth group.

**Land cover: urbanization, agriculture and coastline erosion**

Tourism and coastal urbanization are two intimately linked activities [39]. Urbanization represents the replacement of natural or semi-natural (farmlands) lands by buildings, roads, industries, etc. It avoids the provision of many ecosystem services [40]. Tourism plays an important role in the development of coastal areas; it necessitates important layouts like parking areas and roads located at less than 1 km from the coastline. Tourism developed along the coast causes two types of impact: 1) mass tourism and associated transports and 2) leisure transports. Tourism and urbanization know an exponential increase since 1950 [41]. Similarly, agriculture represents an important part of the potential impacts on the natural and in particular marine environment. [42–44]. Continuously increasing needs in food and energy push agriculture to use fertilizers and pesticides as artificial processes of optimization. Fertilizers brought during inadequate meteorological conditions and/or in quantities superior to what the plants are able to absorb, are taken away by the water and wind to the sea [45]. Eutrophication may then occur and leads to an over-production of phytoplankton and algae. Beyond the visual pollution due to an accumulation of algae on the beaches, it decreases the available oxygen for other aquatic organisms [46]. Similarly, pesticides are found in the marine environment with potential negative impacts on marine fauna and flora but also human health [47,48].

Urbanization and agriculture refer to land use. Different indices measuring anthropogenic pressures undergone by the marine environment use land cover data. Urbanization, agriculture and natural areas or coastal erosion are the most largely used [49] [3]. Data layers concerning urban and agricultural lands within coastal municipalities and on the erosion or aggradation of the coastline were directly extracted under GIS (polylines) from CORINE Land Cover database [50]. 20-m distance matrices are created. The influence on marine waters of urban areas on one part and agricultural lands on another part is assumed to exponentially decrease according to the following equation: *y=99.175e-0.456x* until 10 km. Concerning the coastline erosion, it is assumed to exponentially decrease according to the following equations: *y=99.175e-0.912x* until 5 km for an aggradations and *y=99.175e-1.52x* until 3 km for the erosion. Bathymetry is considered in each model with a theoretical loss of 10 % per 10-m depth group.

**Coastal population density**

Population along the coast involves the presence of consumers, so an increased need in resources (water, energy, raw material) and in natural areas for recreational activities and the emission of varied discards in waters, soils and air [44]. [51] interested in identifying the impacts linked to the nautical frequentation, highlighted the important role of boats (anti-fooling, sacrificial anode, fumes) and the pollutions and nuisance caused by amateur yachtsmen (organic and bacterial pollution *via* direct discards, soaps and detergents, macrowaste, fishing, scuba-diving and spearfishing).

The GIS layer is composed of the different municipalities located within a 5-km radius from the sea. For each municipality, different data are added from the INSEE database ([www.insee.fr](http://www.insee.fr)): the population size and density in 2009, the number of hotel rooms in 2013, the number of campsites in 2013, number of secondary residences in 2010 and the percent of secondary residences in 2013. A note is attributed to each criterion in function of the following categories:

- Population size in 2009: (0) ≤ 500, (1) ]500;2000], (2) >2000 inhabitants
- Population density in 2009: (0) ≤ 10 , (1) ]10;30], (2) ]30;80], (3) ]80;300], (4) ]300;2000], (5) >2000 inhabitants /km²

A 20-m distance matrix is created. An attenuation curve is assumed with a negative exponential decrease from the origin until 1 km for a global note (mean of the notes concerning population density and population size) of 0, 1 or 2 according to *y=99.175e-4.56x*, until 3 km according to y=99.175e-1.52x for a note of 3, until 5 km according to *y=99.175e-0.912x* for a note of 4 and until 20 km according to *y=99.175e-0.228x* for a note of 5. Bathymetry is considered in each model with a theoretical loss of 10 % per 10-m depth group.

**Fishing**

Fishing causes direct and indirect effects on the marine environment. The European Union divides the directs effects into four categories [52]: 1) the impact on the target species and their size, 2) the impact on the non-target species (bycatch + ghost fishing + species depending on fished species), 3) impacts on the habitats and 4) impacts on mammals, birds and other vulnerable species. Indicators have been developed in order to measure the impact of fishing on fish communities.

Rochet and Trenkel [53] showed that the most relevant indicators for management are those linked to the population level (exploitation rate, average catch size, for example), but that several indicators are necessary. The impact of fishing actually depends on the engine type used, the spatio-temporal intensity of the activity, synergistic effects of others activities or the initial complexity level of the physical and biological environment [54]. For example trawling on *P. oceanica* seagrass meadows is recognized as major degradation source especially between -15 and -30 m [55]. Fishing engines may be subdivided into three categories: towed engines (trawling, dredging), passive engines (nets and drift nets, longline and drift longline, handline), and mobile engines (seine, downrigger tracking). Each one focuses on different type of fish and affects the environment by a different way [54,56,57].

The fishing GIS layer contains the localization of traditional and recreational fishing areas after cross referencing based on field data (Andromède océanologie, unpublished data) noting the position of buoy net along the coast and pontoons or groyns concentrating fishing activities, MEDOBS data (aerial pictures, http://www.observatoire-mer.fr/en/autres_pressions.html) localizing buoy nets and fishermen on the sea (unpublished data) and inquiries (data obtained from marine protected areas and managed marine areas). Field data were opportunistically acquired on the occasion of boat campaigns along the coast between 2008 and 2014 (around 100 days per year, 1/3 of the French coast per year). MEDOBS data were acquired between 2011 and 2014 with the help of a georeferenced sophisticated numeric video camera taken on a plane. The data acquisition was led between april and october at a rate of once per week during the summer and once every two weeks in spring and autumn.

A 20-m distance matrix is created. A negative exponential attenuation curve (*y=6E-05x3+0,0034x^2^-0,0583x+3,5699*) is assumed with a decrease from –50 m to –120 m deep.

1. Halpern BS, Walbridge S, Selkoe KA, Kappel C V, Micheli F, D’Agrosa C, et al. A global map of human impact on marine ecosystems. Science. 2008;319: 948–952. doi:10.1126/science.1149345

2. Selkoe KA., Halpern BS, Ebert CM, Franklin EC, Selig ER, Casey KS, et al. A map of human impacts to a “pristine” coral reef ecosystem, the Papahānaumokuākea Marine National Monument. Coral Reefs. 2009;28: 635–650. doi:10.1007/s00338-009-0490-z

3. Micheli F, Halpern BS, Walbridge S, Ciriaco S, Ferretti F, Fraschetti S, et al. Cumulative human impacts on mediterranean and black sea marine ecosystems: assessing current pressures and opportunities. PLoS One. 2013;8: e79889. doi:10.1371/journal.pone.0079889

4. Halpern BS, Selkoe KA, Micheli F, Kappel CV. Evaluating and ranking the vulnerability of global marine ecosystems to anthropogenic threats. Conserv Biol. 2007;21: 1301–15. doi:10.1111/j.1523-1739.2007.00752.x

5. Halpern BS, Walbridge S, Selkoe KA, Kappel CV., Micheli F, D’Agrosa C, et al. A Global Map of Human Impact on Marine Ecosystems. Science (80- ). 2008;319: 948–952. doi:10.1126/science.1149345

6. Meinesz A, Lefevre JR, Astier JM. Impact of Coastal Development on the Infralittoral Zone Along the Southeastern Mediterranean Shore of Continental France. Mar Pollut Bull. 1991;23: 343–347.

7. Meinesz A, Chancollon O, Garcia D, Markovic L. Côtes méditerranéennes françaises, inventaire Observatoire de l’impact des aménagements construits sur la mer. Rapport final. ECOMERS-UNSA. 2010; 2010.

8. Boudouresque CF, Bernard G, Bonhomme P, Charbonnel E, Diviacco G, Meinesz A, et al. Protection and conservation of Posidonia oceanica meadows. RAC/SPA R and, editor. 2012.

9. Ruiz JM, Romero J. Effects of disturbances caused by coastal constructions on spatial structure, growth dynamics and photosynthesis of the seagrass Posidonia oceanica. Mar Pollut Bull. 2003;46: 1523–33. doi:10.1016/j.marpolbul.2003.08.021

10. Montefalcone M, Albertelli G, Morri C, Bianchi CN. Urban seagrass: status of Posidonia oceanica facing the Genoa city waterfront (Italy) and implications for management. Mar Pollut Bull. 2007;54: 206–13. doi:10.1016/j.marpolbul.2006.10.005

11. Milazzo M, Badalamenti F, Ceccherelli G, Chemello R. Boat anchoring on Posidonia oceanica beds in a marine protected area (Italy, western Mediterranean): effect of anchor types in different anchoring stages. J Exp Mar Bio Ecol. 2004;299: 51–62. doi:10.1016/j.jembe.2003.09.003

12. Montefalcone M, Chiantore M, Lanzone A, Morri C, Albertelli G, Nike Bianchi C. BACI design reveals the decline of the seagrass *Posidonia oceanica* induced by anchoring. Mar Pollut Bull. 2008;56: 1637–45. doi:10.1016/j.marpolbul.2008.05.013

13. Marbà N, Duarte CM. Rhizome elongation and seagrass clonal growth. Mar Ecol Prog Ser. 1998;174: 269–280. doi:10.3354/meps174269

14. Almela ED, Marbà N, Álvarez E, Santiago R, Martínez R, Duarte CM. Patch dynamics of the Mediterranean seagrass Posidonia oceanica: Implications for recolonisation process. Aquat Bot. 2008;89: 397–403. doi:10.1016/j.aquabot.2008.04.012

15. Google Earth (Version 7.1.2.2041) [Internet]. 2014. Available: https://www.google.fr/intl/fr/earth

16. Iucn. Guide for the Sustainable Development of Mediterranean Aquaculture. Interaction between Aquaculture and the Environment. [Internet]. Critical Reviews in Environmental Science. 2007. Available: http://www.tandfonline.com/doi/abs/10.1080/10643389109388413

17. Porrello S, Tomassetti P, Manzueto L, Finoia MG, Persia E, Mercatali I, et al. The influence of marine cages on the sediment chemistry in the Western Mediterranean Sea. Aquaculture. 2005;249: 145–158. doi:10.1016/j.aquaculture.2005.02.042

18. Cannac M, Ferrat L, Pergent-Martini C, Pergent G, Pasqualini V. Effects of fish farming on flavonoids in Posidonia oceanica. Sci Total Environ. 2006;370: 91–8. doi:10.1016/j.scitotenv.2006.07.016

19. Holmer M, Argyrou M, Dalsgaard T, Danovaro R, Diaz-Almela E, Duarte CM, et al. Effects of fish farm waste on Posidonia oceanica meadows: synthesis and provision of monitoring and management tools. Mar Pollut Bull. 2008;56: 1618–29. doi:10.1016/j.marpolbul.2008.05.020

20. Delgado O, Ruiz J, Pérez M, Romero J, Ballesteros E. Effects of fish farming on seagrass ( Posidonia oceanica ) in a Mediterranean bay : seagrass decline after organic loading cessation. Oceanol Acta. 1998;22: 109–117.

21. Forchino A, Borja A, Brambilla F, Rodríguez JG, Muxika I, Terova G, et al. Evaluating the influence of off-shore cage aquaculture on the benthic ecosystem in Alghero Bay (Sardinia, Italy) using AMBI and M-AMBI. Ecol Indic. 2011;11: 1112–1122. doi:10.1016/j.ecolind.2010.12.011

22. Marbà N, Santiago R, Díaz-Almela E, Álvarez E, Duarte CM. Seagrass (Posidonia oceanica) vertical growth as an early indicator of fish farm-derived stress. Estuar Coast Shelf Sci. 2006;67: 475–483. doi:10.1016/j.ecss.2005.11.034

23. Ruiz JM, Marco-Méndez C, Sánchez-Lizaso JL. Remote influence of off-shore fish farm waste on Mediterranean seagrass (Posidonia oceanica) meadows. Mar Environ Res. Elsevier Ltd; 2010;69: 118–26. doi:10.1016/j.marenvres.2009.09.002

24. Apostolaki ET, Tsagaraki T, Tsapakis M, Karakassis I. Fish farming impact on sediments and macrofauna associated with seagrass meadows in the Mediterranean. Estuar Coast Shelf Sci. 2007;75: 408–416. doi:10.1016/j.ecss.2007.05.024

25. Bishop MJ, Underwood AJ, Archambault P. Sewage and environmental impacts on rocky shores : necessity of identifying relevant spatial scales. 2002;236: 121–128.

26. Balestri E, Benedetti-Cecchi L, Lardicci C. Variability in patterns of growth and morphology of Posidonia oceanica exposed to urban and industrial wastes: contrasts with two reference locations. J Exp Mar Bio Ecol. 2004;308: 1–21. doi:10.1016/j.jembe.2004.01.015

27. Guilbert A, Holon F, Descamp P. Réalisation de l’état zéro du milieu marin dans la baie de Cavalière. Agence l’eau RMC Publ. 2013; 2013.

28. Guilbert A, Holon F, Descamp P. Suivi écologique du rejet en mer - Station d’épuration des Eaux Blanches - Campagne 2012. Thau Agglomération. 2012;

29. Creocean. Suivi de la station d’épuration MAERA. Bilan 2009-2012. Montpellier Agglomération. 2012;

30. Javel F, Grondin J, Labinal A. Suivi du milieu marin au droit des stations d’épuration d’eaux usées . SAFEGE. Agglomération de Marseille Provence Métropole. 2011;

31. Holon F, Guilbert A, Descamp P. Etablissement de l’état de référence et des modalités de suivi de la qualité du milieu marin – STEP Reyran et Agay –. Communauté d’agglomération Fréjus St Raphaël. 2009;

32. Guilbert A, Holon F, Descamp P. Surveillance du rejet en mer de la station d’épuration, du poste de relevage du Portalet et du bassin de rétention des eaux pluviales de la Ponche – Suivi du milieu naturel. V Saint Tropez. 2013;

33. Guilbert A, Holon F, Descamp P. Suivi de la qualité du milieu marin au droit du rejet des STEP Amphitria, Almanarre, Amphora. Campagne 2012. TPM. 2012; 2012.

34. Holon F, Guilbert A, Freschet E, Deter J. Cartographie évolutive des herbiers de Posidonie en Région PACA sur la période 1922 - 2011. Agence l’eau RMC Publ. 2012;

35. Lafabrie C, Pergent G, Pergent-Martini C. Utilization of the seagrass Posidonia oceanica to evaluate the spatial dispersion of metal contamination. Sci Total Environ. Elsevier B.V.; 2009;407: 2440–6. doi:10.1016/j.scitotenv.2008.11.001

36. Kress N, Herut B, Galil BS. Sewage sludge impact on sediment quality and benthic assemblages off the Mediterranean coast of Israel-a long-term study. Mar Environ Res. 2003;57: 213–33. doi:10.1016/S0141-1136(03)00081-3

37. Agunbiade FO, Awe AA, Adebowale KO. Fuzzy logic-based modeling of the impact of industrial activities on the environmental status of an industrial estate in Nigeria. Toxicol Environ Chem. 2011; 37–41.

38. Jijaescu L. Environmental pollution and global climate change the impact of industrial activity on the environment. Metal Int. 2009;

39. Benoit G, Comeau A. Les perspectives du Plan Bleu sur l’environnement et le développement. Ed l’Aube. 2005;107: 428.

40. Bulleri F, Chapman MG. The introduction of coastal infrastructure as a driver of change in marine environments. J Appl Ecol. 2010;47: 26–35. doi:10.1111/j.1365-2664.2009.01751.x

41. Davenport J, Davenport JL. The impact of tourism and personal leisure transport on coastal environments: A review. Estuar Coast Shelf Sci. 2006;67: 280–292. doi:10.1016/j.ecss.2005.11.026

42. Readman JW, Liong Wee Kwong L, Mee LD, Bartocci J, Nilve G, Rodriguez-Solano J a., et al. Persistent organophosphorus pesticides in tropical marine environments. Mar Pollut Bull. 1992;24: 398–402. doi:10.1016/0025-326X(92)90500-6

43. Tilman D. Global environmental impacts of agricultural expansion: the need for sustainable and efficient practices. Proc Natl Acad Sci U S A. 1999;96: 5995–6000. doi:10.1073/pnas.96.11.5995

44. Savage C, Leavitt PR, Elmgren R. Effects of land use, urbanization, and climate variability on coastal eutrophication in the Baltic Sea. Limnol Oceanogr. 2010;55: 1033–1046. doi:10.4319/lo.2010.55.3.1033

45. FAO. Perspectives pour l’environnement, L'agriculture et l'environnement [Internet]. 2002. Available: http://www.fao.org/docrep/004/y3557f/y3557f11.htm

46. Menesguen A. Eutrophisation des eaux marines et saumâtres en Europe. Rapp IFREMER pour la Comm Eur. 2001;

47. Vagi MC, Petsas a. S, Kostopoulou MN, Karamanoli MK, Lekkas TD. Determination of organochlorine pesticides in marine sediments samples using ultrasonic solvent extraction followed by GC/ECD. Desalination. 2007; doi:10.1016/j.desal.2006.06.020

48. Van der Ooost R, Beyer J, Vermeulen NPC. Bioaccumulation and biomarkers in environmental risk assessment : a review. Environ Toxicol Pharmacol. 2003;

49. Lopez y Royo C, Pergent G. Thèse de Doctorat- Utilisation de Posidonia oceanica ( L .) Delile comme outil de gestion de la qualité écologique du milieu littoral. Univ di Corsica Pasquale Paoli. 2008;

50. SOeS, European Union. CORINE Land Cover [Internet]. 2006. Available: http://www.statistiques.developpement-durable.gouv.fr/donnees-ligne/li/1825.html

51. Peuziat I. Plaisance et environnement. Pratiques, représentations et impacts de la fréquentation nautque de loisir dans les espaces insulaires. Thèse de doctorat. Univ Bretagne Occidentale. 2009;

52. Proceedings of Seas at risk annual conference. Towards sustainable European fisheries : The double challenge of restructuring and reducing the fishing fleet. seas at risk. 2009.

53. Rochet M-J, Trenkel VM. Which community indicators can measure the impact of fishing? A review and proposals. Can J Fish Aquat Sci. Perspective; 2003;60: 86–99. doi:10.1139/f02-164

54. Broeg K. Towards Low Impact Fishery Techniques. WWF Ger Int Cent Mar Conserv. 2008;

55. González-Correa JM, Bayle JT, Sánchez-Lizaso JL, Valle C, Sánchez-Jerez P, Ruiz JM. Recovery of deep Posidonia oceanica meadows degraded by trawling. J Exp Mar Bio Ecol. 2005;320: 65–76. doi:10.1016/j.jembe.2004.12.032

56. Hoskin M. Census of opinions on interactions between fisheries and the environment : a summary report. Invest in Fich South West, Work programme 9: Evaluation of wildlife issues, Coastal and Marine Environmental Research Ltd., Falmouth, UK. ICES. 2006.

57. Chuenpagdee R, Morgan LE, Maxwell SM, Norse EA, Pauly D. Shifting gears : assessing collateral impacts of fishing methods in US waters In a nutshell : Front Ecol Environ. 2003;1: 517–524.
